# Right ventricular cardiomyocyte expansion accompanies cardiac regeneration in newborn mice after large left ventricular infarcts

**DOI:** 10.1172/jci.insight.176281

**Published:** 2024-02-06

**Authors:** Tianyuan Hu, Mona Malek Mohammadi, Fabian Ebach, Michael Hesse, Michael I. Kotlikoff, Bernd K. Fleischmann

**Affiliations:** 1 Institute of Physiology I, Life and Brain Center, Medical Faculty, University of Bonn, Germany.; 2 Department of Neonatology and Pediatric Intensive Care, University Hospital Bonn, Germany.; 3 Department of Biomedical Sciences, Cornell University, Ithaca, New York, USA.

**Keywords:** Cardiology, Cardiovascular disease, Cell cycle

## Abstract

Cauterization of the root of the left coronary artery (LCA) in the neonatal heart on postnatal day 1 (P1) resulted in large, reproducible lesions of the left ventricle (LV), and an attendant marked adaptive response in the right ventricle (RV). The response of both chambers to LV myocardial infarction involved enhanced cardiomyocyte (CM) division and binucleation, as well as LV revascularization, leading to restored heart function within 7 days post surgery (7 dps). By contrast, infarction of P3 mice resulted in cardiac scarring without a significant regenerative and adaptive response of the LV and the RV, leading to subsequent heart failure and death within 7 dps. The prominent RV myocyte expansion in P1 mice involved an acute increase in pulmonary arterial pressure and a unique gene regulatory response, leading to an increase in RV mass and preserved heart function. Thus, distinct adaptive mechanisms in the RV, such as CM proliferation and RV expansion, enable marked cardiac regeneration of the infarcted LV at P1 and full functional recovery.

## Abstract 2

The adaptive response of the right ventricle following left ventricle infarction at postnatal day 1 enables marked cardiac regeneration and full functional recovery.

## Introduction

Large myocardial infarctions (MIs) and ensuing heart failure comprise a significant medical challenge with a poor prognosis, correlating with the lack of regenerative potential of the adult heart. In fact, ^14^C-dating approaches revealed a low cardiomyocyte (CM) turnover rate in adult human hearts, which further decreased with age (1, 2), suggesting an insufficient capacity to replenish lost contractile cells upon injury, resulting in scar formation and adverse remodeling. By contrast, data from human hearts (3) and experimental studies in neonatal rodents and pigs showed that the postnatal heart is capable of partial regeneration in the first days after birth, but that this capacity is lost within a few days (4–7). This is in line with the notion that mammalian CMs retain their proliferative capacity early in development, but this is rapidly lost after birth, after which injury results in hypertrophy and adverse remodeling (8).

An increased rate of CM proliferation triggered by local ischemia has been suggested to be responsible for the regenerative capacity of the neonatal heart, but the underlying molecular mechanisms are still not fully understood and a source of ongoing mechanistic controversy (9–12). Additionally, whether injury results in myocyte cell cycle reentry or a delay in cell cycle exit and enhanced proliferation is unclear (13). Early studies used apex resection or cryoinjury in neonatal mice, but these do not recapitulate the typical pathophysiological features of an ischemic injury and may result in somewhat distinct tissue responses. Specifically, apex resection studies appear to produce minimal fibrosis and a blastema-like outgrowth (4), whereas cryoablation results in significant scarring and only partial regeneration (7). Left anterior descending coronary artery (LAD) ligation studies have become the method of choice, but variable suture positioning and resulting injury size variability have complicated mechanistic studies (14–16).

In order to more fully explore the mechanisms underlying neonatal heart regeneration, we established a large, reproducible ischemic lesion of the left ventricle (LV) in neonatal mice by cauterizing the root of the left coronary artery (LCA). Using this reproducible ischemic injury, we demonstrate that postnatal cardiac regeneration is restricted to a very small time window, as by postnatal day 3 (P3) ischemia does not evoke regenerative cellular responses. Importantly, however, at P1 ischemic injury triggers a significant increase in CM cytokinesis and binucleation throughout the heart, yielding fast and full functional recovery despite persistent scar formation. This response was associated with an increase in pulmonary arterial pressure and a prominent adaptive response of the nonischemic right ventricle (RV), as shown by a striking shift in gene expression, a wave of cardiac myocyte cell cycle reentry, and angiogenesis. The extensive RV cellular expansion increased heart mass, apparently supporting the recovery of heart function. These results establish the importance of stress-induced stimulation of CM division as a critical mechanism in neonatal heart regeneration, and further define the critical role of RV plasticity in LV regeneration.

## Results

### Functional recovery from LV ischemic heart injury involves a prominent adaptive response of the RV.

**Figure 1:**
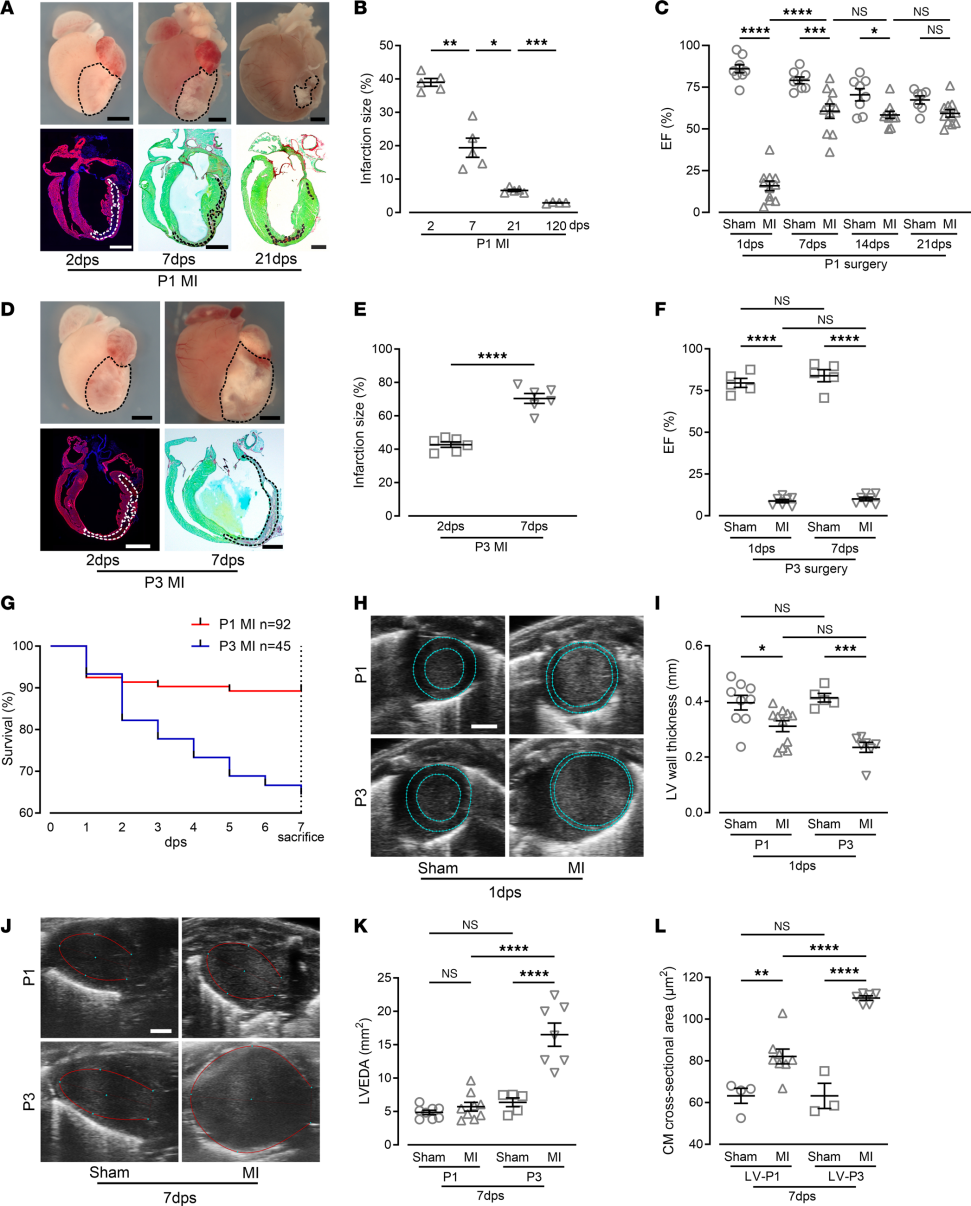
Cauterization of the root of the LCA results in opposite outcomes in P1 and P3 MI mice. (**A**) P1 MI hearts and heart sections at 2, 7, and 21 days post surgery (dps). Visualization of scar areas (marked with dashed lines) with TNNI3 and DAPI or Sirius red and Fast Green costaining. Scale bars: 1 mm. (**B**) Quantitation of infarct size (percentage of the LV). (**C**) Ejection fraction (EF) measured with echocardiography in P1. (**D**) P3 MI hearts and heart sections, visualization of scar area (marked with dashed lines) with TNNI3 and DAPI or Sirius red and Fast Green costaining. Scale bars: 1 mm. (**E**) Quantitation of infarct size. (**F**) EF measured with echocardiography. (**G**) Kaplan-Meier survival curve of mice after P1 MI and P3 MI. (**H**) Echocardiographic short-axis view of MI and sham hearts; dashed lines mark endocardial and epicardial regions. Scale bar: 1 mm. (**I**) Echocardiographic measurements of LV wall thickness in MI and sham hearts. (**J**) Echocardiographic long-axis view of MI and sham hearts; red lines mark the endocardium of the LV at the end of diastole. Scale bar: 1 mm. (**K**) Echocardiographic quantitation of the LV end diastolic area (LVEDA) in MI and sham hearts. (**L**) Quantitation of LV cardiomyocyte (CM) cross-sectional area in MI and sham hearts. *P < 0.05; **P < 0.01; ***P < 0.001; ****P < 0.0001 by unpaired, 2-tailed Student’s *t* test (**E**) or 1-way ANOVA with Holm-Šidák post hoc test (**B**, **C**, **F**, **I**, **K**, and **L**). Data in **G** were evaluated with the log-rank test: *P* = 0.0006. NS, no significance.

In order to create consistent, ischemic heart lesions in neonatal mice, we used a small cauterizer to ablate the root of the LCA in P1 (P1 MI) mice (Supplemental Figure 1A; supplemental material available online with this article; https://doi.org/10.1172/jci.insight.176281DS1). This produced a large, highly reproducible ischemic injury in the LV, with recovery rates above 90%. At 2 days post surgery (dps), large parts of the LV of P1 MI mice were injured, as shown by lack of troponin I (TNNI3) staining (Figure 1A). The lesion typically affected the middle layer of the myocardium, leaving uninjured inner and outer layers of myocardium and intact endocardial and epicardial layers (Figure 1A and Supplemental Figure 1B). The injury size was very large at 2 dps (38.99% ± 1.18% of the LV, *n* = 5), decreased significantly (19.40% ± 2.88%, *n* = 5) at 7 dps, and was minimal at 21 dps (6.59% ± 0.33%, *n* = 5) (Figure 1B). Functional assessment of the LV with echocardiography showed a strong reduction in the LV ejection fraction (EF) and fractional shortening (FS) at 1 dps compared with shams, which was associated with an increase in LV end-diastolic area (LVEDA) (Figure 1C and Supplemental Figure 1, C and D). Importantly, LV function almost completely recovered at 7 dps in P1 MI mice, and functional measurements were indistinguishable from shams at 14 and 21 dps (Figure 1C and Supplemental Figure 1, C and D).

By contrast, cauterization of P3 mice, in which a more adult-like compacted myocardium has developed (17), resulted in an enlarging lesion and loss of function (Figure 1, D–F). Lesion size in P3 MI 2-dps hearts was similar to P1 MI hearts (42.74% ± 1.64%, *n* = 6; Figure 1, B and E, and Supplemental Figure 1, B and E), but increased in size at 7 dps and became transmural (70.41% ± 2.99%, *n* = 6; Figure 1, D and E). Moreover, LV function remained compromised, often leading to spontaneous death or requiring euthanasia (Figure 1G and Supplemental Figure 1F). Echocardiography indicated a similar reduction in LV wall thickness, and an increase in LVEDA at 1 dps in P1 MI and P3 MI mice (Figure 1, H and I, and Supplemental Figure 1G), whereas at 7 dps both parameters had recovered in P1 MI, but not in P3 MI hearts (Figure 1, J and K, and Supplemental Figure 1H), and CM cross-sectional area was 40% higher in P3 mice at 7 dps, indicating hypertrophy and adverse remodeling (Figure 1L and Supplemental Figure 1I).

**Figure 2:**
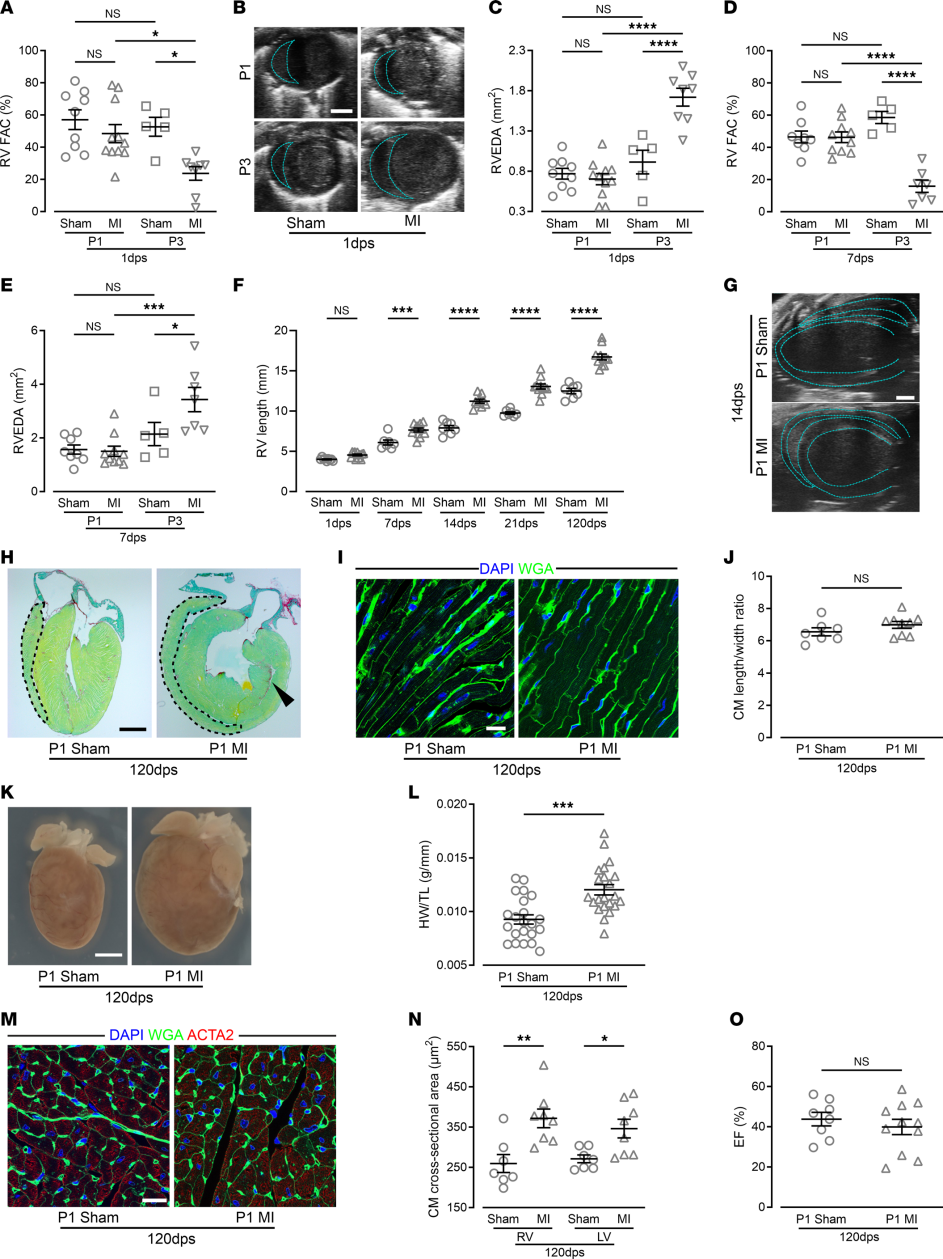
RV function and morphology differ between P1 MI and P3 MI hearts; P1 MI hearts display a small persistent scar and adaptive hypertrophy at 120 dps. (**A**) Echocardiographic measurement of RV fractional area change (RV FAC). (**B**) Echocardiographic short-axis view of the ventricles; dashed blue lines indicate the RV end diastolic area (RVEDA). Scale bar: 1 mm. (**C**–**E**) Echocardiographic measurements of RVEDA (**C** and **E**) and RV FAC (**D**). (**F**) Echocardiographic measurements of the length of the RV free wall after P1 surgery. (**G**) Echocardiographic longitudinal axis view of P1 ventricles; blue lines indicate endo- and epicardial layers of the RV and the LV. Scale bar: 1 mm. (**H**) Heart sections of a P1 MI and sham heart; black dashed lines mark the RV free wall. The RV is elongated in the P1 MI heart. Black arrowhead indicates residual scar. Scale bar: 2 mm. (**I** and **J**) Heart sections (**I**) and quantitation (**J**) of length/width ratio of CMs costained with WGA and DAPI in the RV. Scale bar: 10 μm. (**K** and **L**) Images (**K**) and quantitation (**L**) of heart weight (HW)/tibia length (TL) ratio. Scale bar: 2 mm. (**M** and **N**) Heart sections stained for α-actin (ACTN2) and with WGA and DAPI (**M**), and quantitation of CM cross-sectional area (**N**) in both ventricles. Scale bar: 20 μm. (**O**) LV EF. *P < 0.05; **P < 0.01; ***P < 0.001; ****P < 0.0001 by 1-way ANOVA with Holm-Šidák post hoc test (**A**–**F**) or unpaired, 2-tailed Student’s *t* test (**J**, **L**, and **O**). NS, no significance.

Surprisingly, we observed a prominent difference in the immediate functional response of the RV to MI in P1 MI and P3 MI mice when examining RV function through echocardiographic measurements of fractional area change and RV end-diastolic area. These measurements maintained function in the RV of P1 MI mice (Figure 2, A–E). This correlated with major morphological changes in the RV in P1 MI mice, as it became more elongated at 7 dps and increased further with age (Figure 2F). Moreover, echocardiography revealed that the RV even partially wrapped around the LV and formed the tip of the heart at 14 and 21 dps (Figure 2G and Supplemental Videos 1–6). An elongation of the RV was also found in histological sections of P1 MI hearts and a steady increase up to 120 dps occurred (Figure 2, F and H), but the RV CM length/width ratio remained very similar in MI and sham mice (Figure 2, I and J). In contrast to P1 MI mice, echocardiography revealed deterioration of RV function and global heart failure in P3 MI mice at 1 and 7dps (Figure 2, A–E). These data identified prominent adaptive changes in the RV that underlie the functional rescue from injury in P1 MI, but not P3 MI, mice.

### LV and vascular adaption to left heart ischemia.

To determine the extent of regeneration, we examined the hearts of P1 MI mice at 120 dps and detected only a small remaining scar in the LV (2.92% ± 0.10% of total LV area, *n* = 4) (Figure 2H). At this stage, the hearts displayed a roundish shape (Figure 2K). Heart weight/tibia length, heart weight/body weight ratios, and the average cross-sectional area of CMs in both ventricles were all significantly increased, consistent with an increase in heart cell mass (Figure 2, L–N, and Supplemental Figure 2A). However, no signs of pathological remodeling such as interstitial fibrosis were found, and EF, cardiac output (CO), and LVEDA were similar to those of sham hearts (Figure 2O and Supplemental Figure 2, B–D).

**Figure 3:**
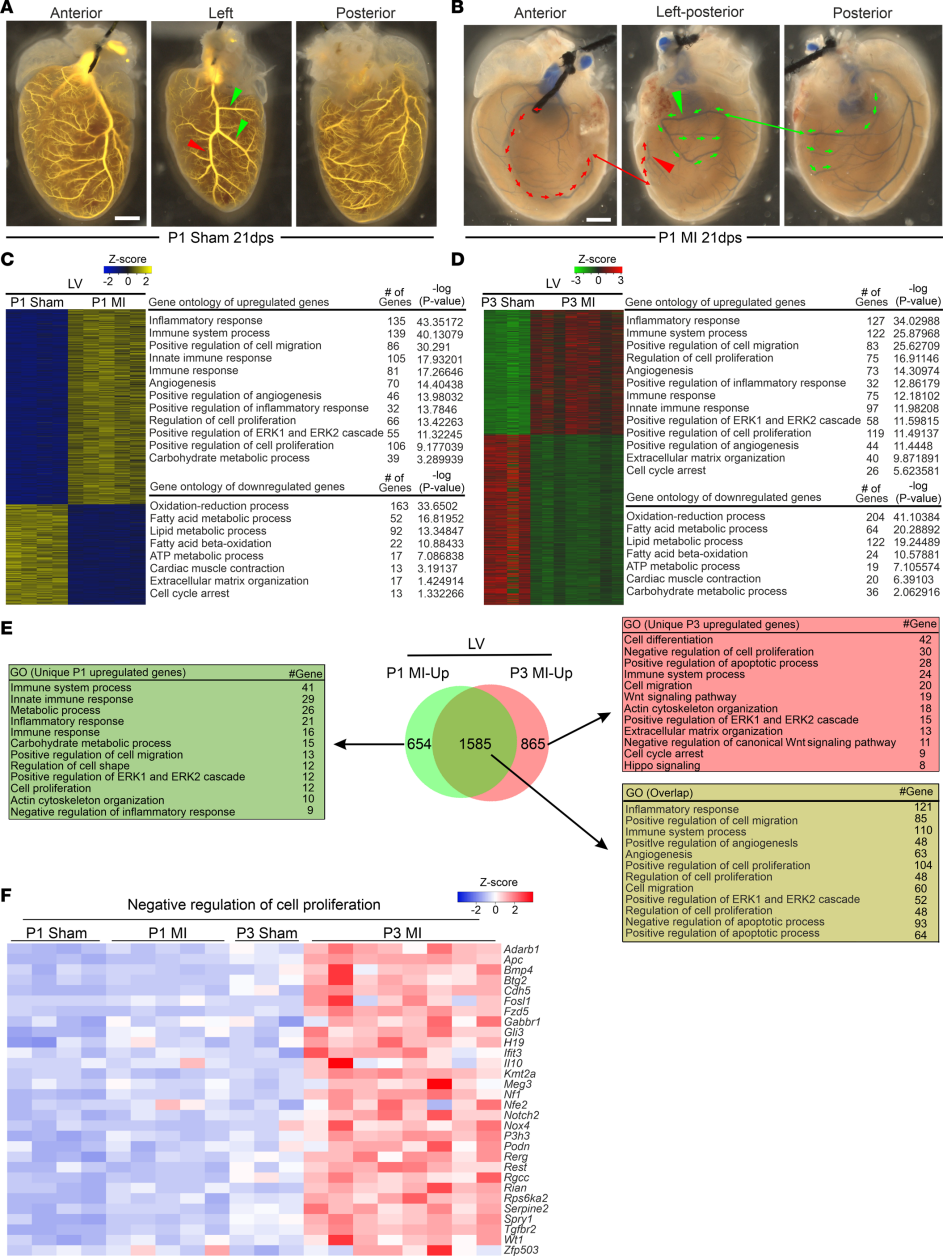
Atypical revascularization in P1 MI hearts; transcriptome analysis of the LV at 1 dps reveals a large overlap, but also striking differences between P1 MI and P3 MI compared with sham hearts. (**A** and **B**) Coronary artery vessel casting: red arrowheads mark LAD coronary artery or the residual of it after MI, green arrowheads left circumflex coronary artery or the residual of it after MI, red arrows indicate the direction of the blood flow from the septal coronary artery to the LV after MI, and green arrows indicate the direction of the blood flow from the right coronary artery to the LV after MI. Scale bar: 1 mm. (**C** and **D**) Heatmaps of differentially regulated genes and gene ontology (GO) of biological processes of upregulated and downregulated genes in the LV. (**E**) Venn diagram showing a high overlap in genes overexpressed in the LV; tables list selected uniquely and overlapping GOs of upregulated genes in LV. (**F**) Heatmap of genes related to biological processes of “negative regulation of cell proliferation” in LV.

Regenerative adaptation to ischemic infarction in P1 mice was also associated with substantial vascular remodeling. Vessel casting at 21 dps demonstrated an atypical septal coronary artery (SCA), which was elongated and extended toward the ischemic area in the LV in 86% of hearts (*n* = 29) (Figure 3, A and B, and Supplemental Figure 2E). Other vascular adaptations included elongation and expansion of the right coronary artery (RCA) to the left side of the heart (48.3%; Supplemental Figure 2F). In some hearts, vascular connections to the lung and the chest wall (27.5%) or the coronary vein (13.79%) were observed (Supplemental Figure 2, G and H).

### LV neonatal cardiac regeneration.

RNA-seq analysis of the LV identified activation of distinct and highly overlapping (1585 genes, 70%) gene expression patterns in P1 and P3 MI hearts, including genes previously associated with pathologic hypertrophy (Figure 3, C–E) (18). These comprised gene categories identified by positive regulation of inflammatory responses, cell migration, angiogenesis, cell proliferation, and the ERK1 and ERK2 signaling cascade. However, important differences in P1 MI and P3 MI cardiac gene expression were also noted, as genes associated with positive regulation of apoptosis, cell cycle arrest, and Hippo signaling, and negative regulation of cell proliferation and canonical Wnt signaling were unique for P3 MI LVs (Figure 3, E and F). Interestingly, RNA-seq data of the septum showed that the gene expression pattern in P1 MI and P3 MI hearts was very similar to that in the LV (Supplemental Figure 3, A–C).

**Figure 4:**
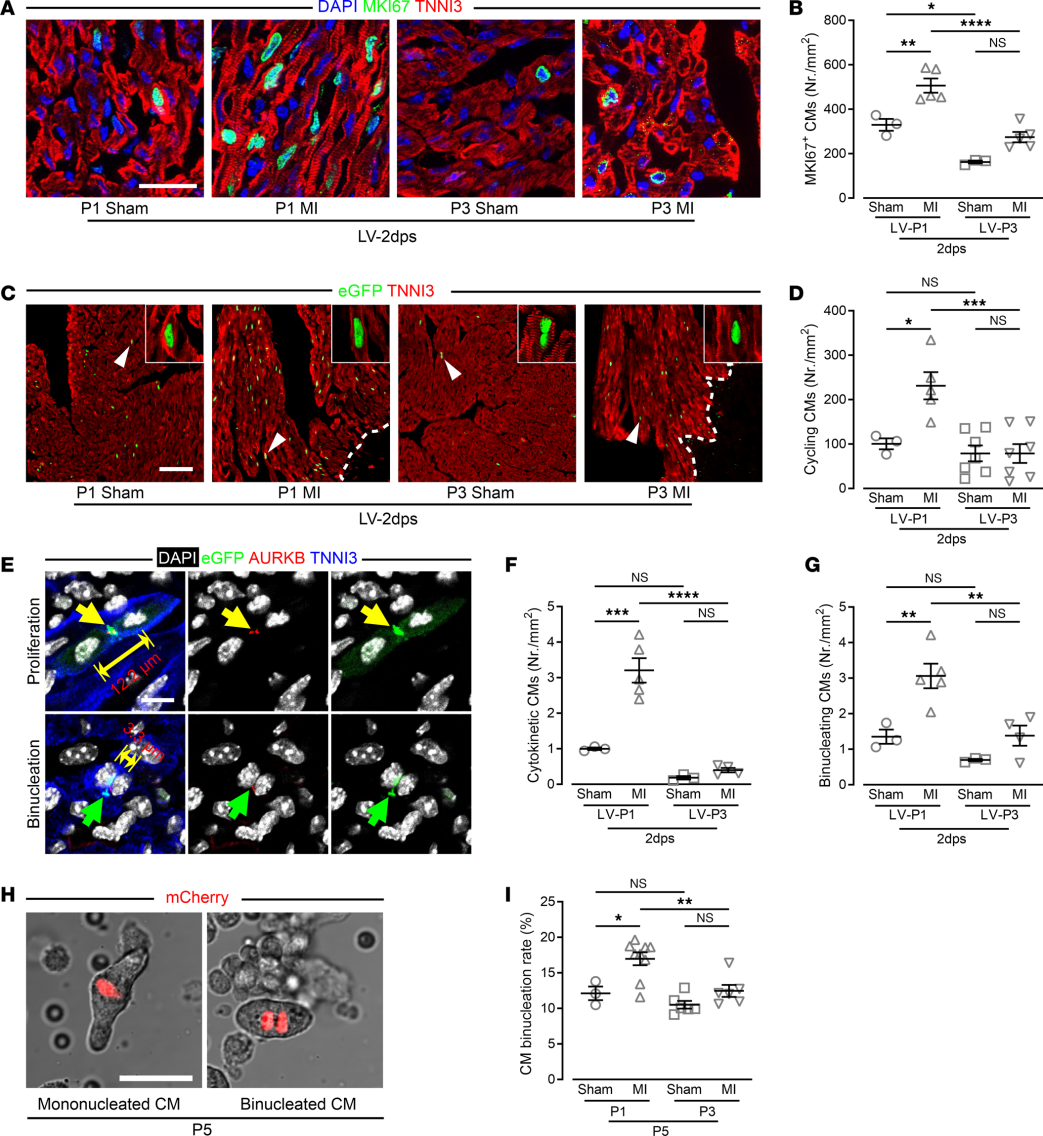
CM cytokinesis and binucleation strongly increase in the LV of P1, but not of P3, MI hearts. (**A** and **B**) Images (**A**) and quantitation (**B**) of MKI67^+^ CMs in LV marked by costaining for MKI67, TNNI3, and with DAPI. Scale bar: 20 μm. (**C**) Mosaic images of LV heart sections of *CAG-eGFP-anillin* mice costained for eGFP and TNNI3; arrowheads mark eGFP^+^ CMs, which are shown at higher magnification (×5.5) as confocal images in insets. Scale bar: 20 μm. (**D**) Quantitation of eGFP^+^ CMs in the LV. (**E**–**G**) Cytokinetic and binucleating CMs in heart sections costained for eGFP, AURKB, TNNI3, and with DAPI; midbodies were identified based on costaining for eGFP and AURKB (arrows). Two-sided arrows mark the distance between the 2 nuclei in the same CM; typical (yellow arrows) or atypical midbody location (green arrows) mark cytokinetic (**F**) and binucleating (**G**) CMs. Scale bar: 10 μm. (**H**) Images of mono- and binucleated CMs isolated from *αMHC-H2BmCherry* hearts at P5. Scale bar: 20 μm. (**I**) Quantitation of the percentage of binucleated/total CMs isolated from whole hearts. *P < 0.05; **P < 0.01; ***P < 0.001; ****P < 0.0001 by 1-way ANOVA with Holm-Šidák post hoc test. NS, no significance.

Because of the prominent upregulation of genes involved in cell proliferation and apoptosis in the LV of P1 MI hearts, we further investigated postinfarction CM proliferation. Increased cell cycle activity was indicated by significantly enhanced MKI67 staining in the LV of P1 MI hearts (Figure 4, A and B, and Supplemental Figure 4A), and cell division was confirmed using transgenic *CAG-eGFP-anillin* mice in which cells display cell cycle–specific cytosolic sublocalization of eGFP (19, 20). Infarction of P1 *CAG-eGFP-anillin* mice resulted in a 2-fold increase in eGFP-ANILLIN^+^/TNNI3^+^ interphase CMs in LV heart sections, whereas no increase was found in the LV of P3 MI hearts (Figure 4, C and D, and Supplemental Figure 4B). Finally, using midbody position analysis (19), we found that the number of CMs undergoing cytokinesis and binucleation in the LV of P1 MI hearts was more than 2-fold higher than in the LV of P1 shams, whereas neither cytokinesis nor binucleation was significantly increased in the LV of P3 MI hearts (Figure 4, E–G). Increased CM binucleation rates relative to P3 MI hearts were confirmed in CMs isolated from P1 MI 4-dps and P3 MI 2-dps *αMHC-H2BmCherry* mice (21), in which all CM nuclei are marked by red fluorescence (Figure 4, H and I). Thus, infarction at P1 causes CMs to proliferate and binucleate at an increased rate in the LV of P1 MI, but not P3 MI, hearts.

**Figure 5:**
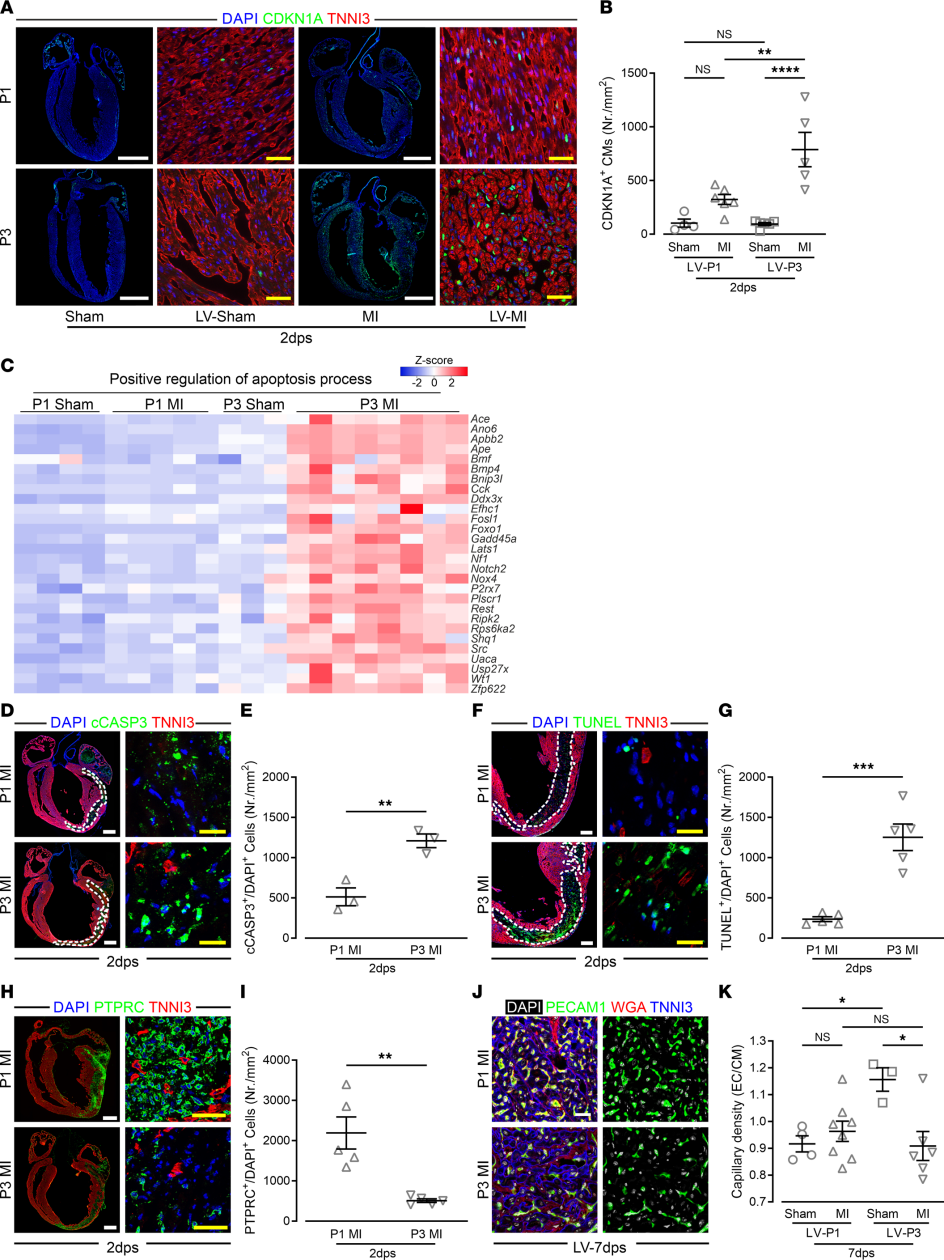
Increased rates of apoptosis and CDKN1A^+^ CMs in the LV of P3 MI compared with P1 MI hearts. (**A**) Mosaic whole-heart and magnified LV images of heart sections costained for CDKN1A, TNNI3, and with DAPI. (**B**) Quantitation of CDKN1A^+^ CMs in heart sections. White scale bars: 1 mm; yellow scale bars: 40 μm. (**C**) Heatmap showing expression of genes related to GO “positive regulation of apoptosis process” in LV. (**D**) Mosaic whole-heart and magnified infarcted-area images of heart sections costained for cleaved caspase 3 (cCASP3), TNNI3, and with DAPI; dashed lines mark infarct areas. White scale bars: 500 μm; yellow scale bars: 20 μm. (**E**) Quantitation of cCASP3^+^ and DAPI^+^ cells in the infarct area. (**F**) Mosaic whole-heart and magnified infarcted-area images of heart sections costained for TUNEL, TNNI3, and with DAPI; dashed lines mark infarct area. White scale bars: 200 μm; yellow scale bars: 20 μm. (**G**) Quantitation of TUNEL^+^ and DAPI^+^ cells in the infarct area. (**H**) Mosaic whole-heart and magnified infarcted-area images of heart sections costained for PTPRC, TNNI3, and with DAPI. White scale bars: 500 μm; yellow scale bars: 40 μm. (**I**) Quantitation of PTPRC^+^ and DAPI^+^ cells in the infarct area. (**J**) Capillaries in LV heart sections costained for PECAM1, TNNI3, and with WGA. Scale bar: 10 μm. (**K**) Quantitation of capillary density assessed by number of PECAM1^+^ cells per CM. *P < 0.05; **P < 0.01; ***P < 0.001; ****P < 0.0001 by 1-way ANOVA with Holm-Šidák post hoc test (**B** and **F**) or unpaired, 2-tailed Student’s *t* test (**E**, **G**, and **I**).

Cyclin-dependent kinase inhibitor 1A (*Cdkn1a*) was significantly upregulated in LV P3 hearts in RNA-seq analysis and CDKN1A staining (Figure 5, A and B), and expression of the upstream *Cdkn1a* signaling elements *Foxo1* and *Akt1* were consistent with cyclase-dependent kinase inhibition limiting cell cycle progression in P3 MI hearts (Supplemental Figure 4C). In addition to inhibition of cell cycle progression, FOXO1 has been implicated in caspase-mediated apoptosis (22), and P3 MI hearts evidenced upregulation of genes related to programmed cell death (Figure 5C). Accordingly, apoptosis rates in the lesion site of P3 MI increased at least 2-fold compared with P1 MI hearts, as assessed by cleaved caspase 3 (cCASP3) staining and terminal deoxynucleotidyl transferase dUTP nick-end labeling (TUNEL), indicating that apoptosis was a prominent feature of the MI response in P3 hearts (Figure 5, D–G). Conversely, immune response genes were upregulated in the injured area of P1 MI hearts, which persisted over time, and PTPRC^+^ cell invasion was increased 4-fold in P1 MI compared with P3 MI hearts (Figure 5, H and I, and Supplemental Figure 4D). P1 MI and P3 MI hearts were also distinguished by the expression of an angiogenesis gene program, and capillary density was reduced by almost 25% in the LV of P3 MI hearts at 7 dps, whereas P1 MI hearts showed no reduction in capillary density (Figure 5, J and K). These data suggest that in the LV the absence of CDKN1A-mediated inhibition of cell cycle progression and programmed cell death, as well as increased cell invasion and angiogenesis, underlie the maintenance of LV post-MI function at P1.

### RV CM expansion and adaption are triggered by LV infarction.

**Figure 6:**
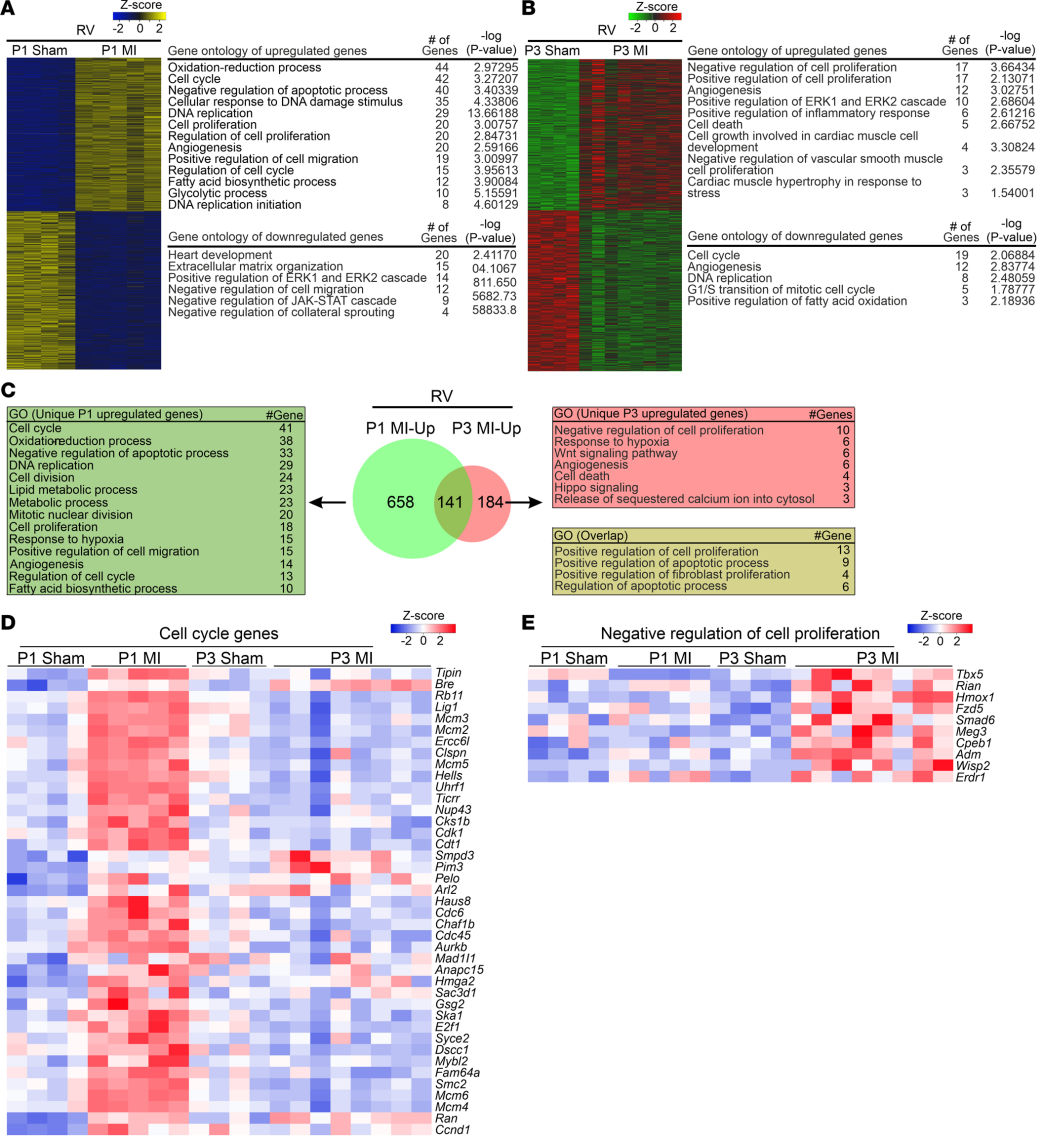
Transcriptomic analysis of the RV in P1 MI, P3 MI, and sham hearts at 1 dps reveals a unique gene expression pattern. (**A** and **B**) Heatmaps show differentially regulated genes and GO of biological processes of upregulated and downregulated genes in the RV. (**C**) Venn diagram depicting uniquely overexpressed genes in the RV, and overlapping genes compared to sham. Tables list selected GO biological processes. (**D** and **E**) Heatmaps showing gene expression in the GO “cell cycle” (**D**) and “negative regulation of cell proliferation” (**E**) in the RV.

**Figure 7:**
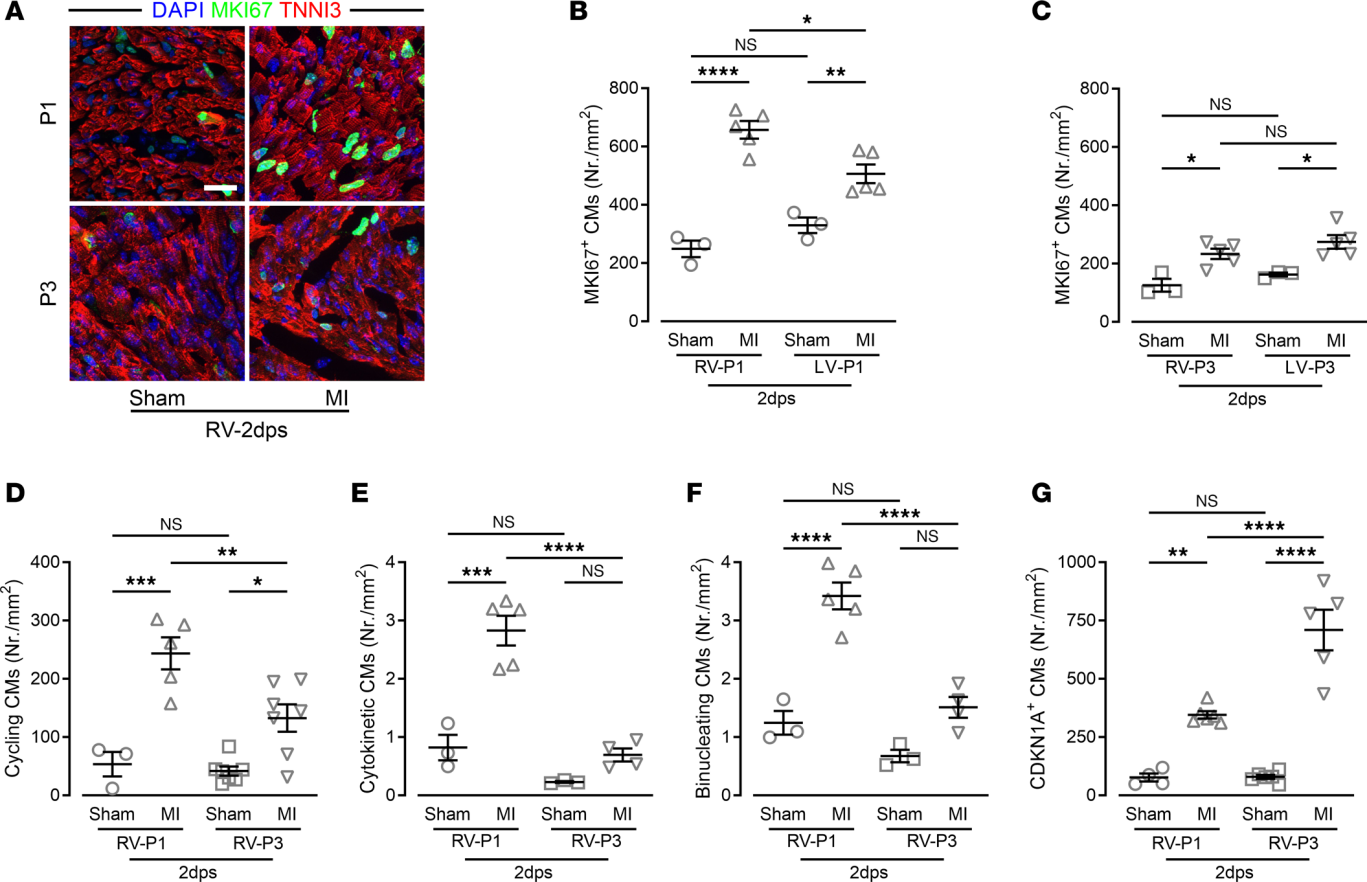
Increased CM proliferation in the RV free wall of P1, but not of P3, MI hearts. (**A**) Confocal images of RV heart sections costained for MKI67, TNNI3, and with DAPI. Scale bar: 20 μm. (**B** and **C**) Quantitation of MKI67^+^ CMs in the RV and LV. (**D**) Quantitation of cycling CMs (eGFP^+^) in the RV of *CAG-eGFP-anillin* hearts. (**E** and **F**) Quantitation of CMs undergoing cytokinesis (**E**) and binucleation (**F**) in the RV of *CAG-eGFP-anillin* hearts. (**G**) Quantitation of CDKN1A^+^ CMs in the RV. *P < 0.05; **P < 0.01; ***P < 0.001; ****P < 0.0001 by 1-way ANOVA with Holm-Šidák post hoc test. NS, no significance.

Unlike the highly overlapping gene expression pattern (70%) in the LV and septum of P1 MI and P3 MI mice, we found in the RV an overlap of only 17% in upregulated genes between P1 MI and P3 MI hearts (Figure 6, A–C). The number of uniquely upregulated genes in the RV of P1 MI hearts (total 799 genes, 658 unique to P1) was more than 2-fold greater than in P3 MI hearts (total 325 genes, 184 unique to P3), indicating a global cardiac response underlying neonatal heart regeneration. For instance, cell cycle–related genes were uniquely upregulated in the RV of P1 MI mice, whereas genes related to negative regulation of cell proliferation characterized the expression pattern of the RV of P3 MI mice (Figure 6, D and E). MKI67^+^ CMs and, to a lesser degree, MKI67^+^ non-CMs, were highly increased in the RV relative to the LV of P1 MI mice (Figure 7, A and B, and Supplemental Figure 5A), whereas MKI67^+^ CMs were fewer in the RV of P3 MI hearts and more similar to the level found in the LV of P3 MI hearts (Figure 7C). Analysis of eGFP-ANILLIN^+^ CMs in RV of P1 MI hearts confirmed a strong increase in CM division and binucleation rates, unlike in P3 MI hearts (Figure 7, D–F, and Supplemental Figure 5, B–D). Consistent with the critical role of cyclin-dependent kinase inhibition in heart regeneration, CDKN1A^+^ CMs were strongly increased in the RV of P3 MI hearts (Figure 7G and Supplemental Figure 5E).

**Figure 8:**
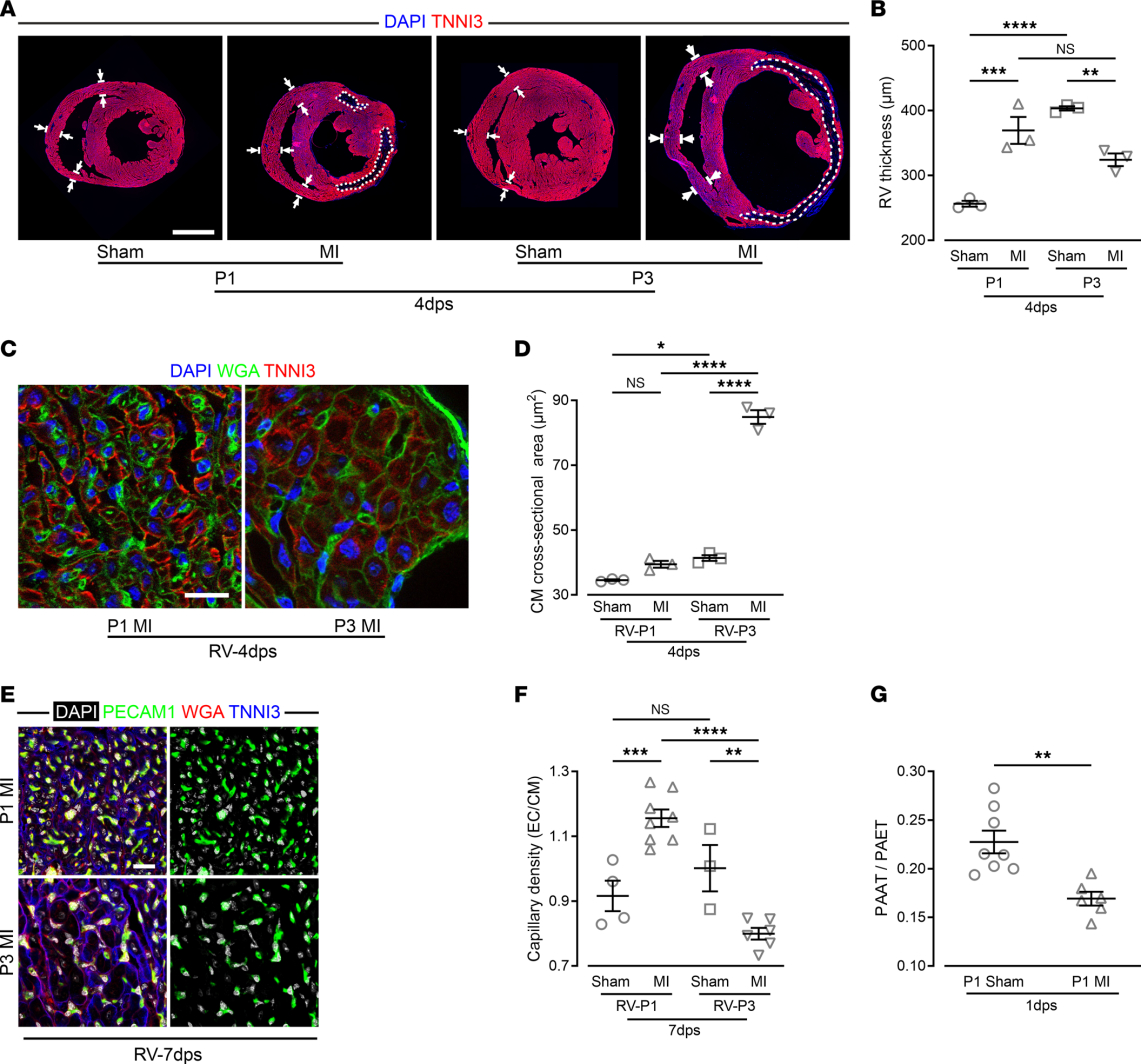
Different adaptive response of the RV of P1 MI and P3 MI hearts, and functional evidence of pulmonary arterial hypertension in P1 MI mice. (**A** and **B**) Images (**A**) and quantitation (**B**) of the wall thickness of heart sections stained for TNNI3 and costained with DAPI; dashed lines mark infarct area, double arrows indicate measuring sites. Scale bar: 1 mm. (**C** and **D**) RV heart sections stained for TNNI3 and costained with WGA and DAPI showing CM sizes (**C**) and quantification (**D**) of CM cross-sectional area in the RV. Scale bar: 20 μm. (**E** and **F**) RV heart sections costained for PECAM1, TNNI3, and with WGA and DAPI showing capillaries (**E**) and quantitation of capillary density (**F**). Scale bar: 20 μm. (**G**) Pulmonary artery acceleration time/pulmonary artery ejection time (PAAT/PAET) ratio obtained with Doppler measurements of flow velocity in the main pulmonary artery in P1 MI and sham mice. *P < 0.05; **P < 0.01; ***P < 0.001; ****P < 0.0001 by 1-way ANOVA with Holm-Šidák post hoc test (**B**, **D**, and **F**) or unpaired, 2-tailed Student’s *t* test (**G**). NS, no significance.

These changes were consistent with prominent histological changes in the RV in P1 MI and P3 MI hearts. The RV wall was over 40% thicker than sham controls in P1 MI hearts, whereas it was 20% thinner in P3 MI hearts at 4 dps (Figure 8, A and B), further suggesting a major role of the RV in the functional rescue. Myocyte hypertrophy was not a feature of RV rescue of infarction, as RV CM cross-sectional area was unaltered in P1 MI hearts, but more than doubled in P3 MI hearts (Figure 8, C and D). In addition, capillary density was increased in the RV of P1 MI hearts at 7 dps, consistent with an upregulation of angiogenic genes, but decreased in the RV of P3 MI hearts (Figure 6, A–C, and Figure 8, E and F). Thus, LV ischemia evokes global cellular responses and prominent myocyte expansion in the RV, which are sufficient to rescue cardiac function in P1, but not P3, mice. However, unlike the ischemic LV, the RV lacks the direct effects of cell death, blood extravasation, inflammatory cell influx, and cytokine release (see also Supplemental Figure 4D). We postulated that postcapillary pulmonary arterial hypertension secondary to LV failure immediately after MI could likely be responsible for such prominent RV adaptation in P1 hearts (Figure 1C). With high-resolution echocardiographic assessment, as recently described for neonatal mice and human infants (23, 24), pulmonary artery acceleration time (PAAT) and pulmonary artery ejection time (PAET) were measured at 1 dps to assess increases in pulmonary arterial pressure (Supplemental Figure 5, F and G). Both PAAT and the PAAT/PAET ratio were significantly decreased in P1 MI mice compared with sham controls, consistent with an increase in pulmonary arterial pressure (Figure 8G and Supplemental Figure 5H).

Transcriptome analysis further indicated that after infarction, the RV in P1 hearts shifts to a gene expression pattern similar to the noninfarcted LV of P1 sham hearts (Supplemental Figure 5, I and J), suggesting that upon LV injury, systemic signals of cardiac stress trigger a critical shift in the expression program associated with a higher end-diastolic pressure. This response was absent in P3 MI hearts, which displayed typical features of a failing RV, indicating the narrow developmental window in which regenerative capacity and functional adaptation are available.

## Discussion

We have established an ischemic injury model in the neonatal mouse heart with a highly reproducible lesion of approximately 40% of the LV, facilitating analysis of the molecular and functional aspects of cardiac regeneration and repair (5, 21). We used this model to demonstrate that large ischemic lesions induced at P1 are almost fully repaired through cellular expansion, with the exception of a small scar that persists to adulthood, and that this regenerative capacity is lost by P3, resulting in heart failure and demise within 7 dps. Surprisingly, LV infarction at P1 triggers a prominent cellular expansion of the RV, and previous studies that have focused on LV regeneration appear to belie the critical role of injury-induced RV myogenesis. Moreover, the stimulation of RV heart cell expansion cannot be explained by the mechanisms of neonatal regeneration advanced to date, as the RV does not experience tissue damage, is not made ischemic by LV infarction, and does not undergo an influx of inflammatory cells. Furthermore, while LV infarction triggers cytokinesis in both the LV and RV, the RV undergoes a unique shift in gene expression characteristic of cell cycle activation.

To determine whether neonatal heart regeneration of the LV is due to CM proliferation, or rather accelerated binucleation resulting in a compensatory hypertrophic response (4, 25, 26), we examined the response in *CAG-eGFP-anillin* mice (19, 26). We show that both CM cytokinesis and binucleation are strongly increased in the LV of P1 MI hearts and hence underlie the prominent regenerative response, whereas they are missing in the LV of P3 MI hearts (Figure 4, F and G). Transcriptome analysis indicated marked differences between the LV of P1 MI and P3 MI hearts regarding gene categories thought to play a key role in neonatal cardiac plasticity (27–29). Consistent with a lack of CM regeneration in the LV of P3 MI hearts, we detected upregulation of signaling pathways such as Hippo and CDKN1A as well as upstream signaling elements, which arrest CM cell cycle activity and induce terminal differentiation (30–33). CDKN1A protein expression was strongly upregulated and apoptosis increased in P3 MI hearts, consistent with the observed exit of CMs from the cell cycle before mitosis and subsequent cellular loss. Vascularization and angiogenesis are thought to be important factors contributing to the regenerative capacity of neonatal hearts (34). Vascular plasticity was a prominent feature of P1 MI hearts, including an atypical and elongated SCA and/or RCA, which extended toward the LV as alternative routes of blood supply, and a return to normal capillary density in the LV by 7 dps, similar to recent studies (34, 35). By contrast, capillary density of the LV was reduced in P3 MI mice. Consistent with the regenerative and angiogenic failure in P3 MI mice, echocardiography revealed LV dilation and CM hypertrophy in P3 MI mice. Moreover, whereas LV function strongly declined after the injury in P1 MI mice, it recovered within 1 week in P1 MI mice, but not in P3 MI mice, resulting in typical signs of heart failure, namely LV dilation and CM hypertrophy.

It remained unclear how P1 MI mice could tolerate the substantial loss of myocardial mass and how LV function is compensated in the immediate aftermath of infarction. Our data indicate that the cellular expansion of the RV likely plays a critical role in this compensation. The RV of P1 MI hearts displayed great plasticity, as it extended in echocardiography and histomorphologically in the longitudinal axis. In the echocardiography the RV even often formed the tip of the heart and wrapped around the LV (Figure 2, G and H, and Supplemental Videos 1–6). This could contribute to the improved LV function observed in P1 MI mice, as the overall drop in EF and CO immediately after the lesion was less pronounced than in P3 MI mice. In these mice, RV wall thickness was reduced and the CMs strongly hypertrophied, consistent with maladaptive remodeling. Capillary density was strongly increased in the RV of P1 MI mice compared with sham controls, suggesting that the prominent regenerative response is closely intertwined with the vascular response, whereas inflammation does not appear to play an important role, as PTPRC^+^ cells were not increased in the RV of P1 MI hearts. A unique gene expression program underlies these responses. Cardiac genes involved in CM proliferation and angiogenesis were strongly upregulated in the RV of P1 MI mice, and analysis of eGFP-ANILLIN^+^ CMs confirmed that the increase in proliferating CMs in the RV free wall was as pronounced as in the injured LV, resulting in a thickened RV heart wall but no CM hypertrophy. In contrast, this adaptive response was entirely missing in the RV of P3 MI hearts. These data show that the RV undergoes a unique adaptive response upon LV injury. We are not aware that this aspect has been investigated in earlier work, as preferentially the response of the entire remote myocardium was analyzed (36). This response is likely limited by a wave of cell cycle exit and/or binucleation that occurs by P3, providing further support for the notion that neonatal myogenesis occurs through an expansion of cell-division-competent myocytes, as opposed to cell cycle reentry.

Thus, a large LV injury induces in the neonatal mouse heart a global regenerative response. The functional data imply that regeneration of the LV in P1 MI mice critically depends on the plasticity and adaptive response of the RV. This is reminiscent of human infants suffering from congenital or acquired disorders of the LV, in which RV function is a key determinant for survival (37, 38). In addition, banding of the pulmonary artery and increase in RV mass is a relatively new therapeutic strategy to improve heart function in infants suffering from LV dilated cardiomyopathy with low-output failure (39, 40). In more general terms, regardless of the age and type of the LV defect, RV dysfunction is known to be a critical clinical parameter contributing to the poor prognosis and high mortality of patients with severe LV heart failure (41). The demonstration of marked myogenesis in the RV of P1 MI mice suggests, as mentioned above, mechanisms and signals other than those presumed to stimulate LV regeneration. Pulmonary arterial hypertension is an immediate consequence of LV failure, and subsequent RV failure has a strong impact on disease progression, morbidity, and mortality (41, 42). We therefore thought that this could be the stressor inducing RV cell expansion and adaptation upon LV cauterization in P1 MI mice. Using echocardiography, we confirmed a significant lowering of PAAT and PAAT/PEAT values, as expected in case of pulmonary arterial hypertension. We propose that the pulmonary arterial hypertension following neonatal P1 LV injury induces an immediate angiogenic and myoproliferative response in the RV, as an abundant population of myocytes has not yet exited the cell cycle. A strategy to further test this hypothesis would be the selective induction of pressure overload in the RV of P1 mice, for instance by pulmonary artery banding without LV injury. Such a surgical model, which has not yet been reported in the literature for neonatal mice, could further corroborate that the pressure increase in the RV is inducing the adaptive response of the RV. This could prove helpful for a better understanding of mechanisms governing cardiac regeneration and the identification of potentially relevant therapeutic targets.

## Methods

### Sex as a biological variable.

Both sexes were involved in the study and sex was not considered as a biological variable.

### Housing and husbandry of mice.

Mice were kept in the animal facility of the University Clinics following the Federation of European Laboratory Animal Science Associations (FELASA) guidelines. Mice were housed on a 12-hour light/dark cycle and had ad libitum access to food and water. A certified veterinarian was assigned to monitor the sanitation of the facility and the health status of the mice.

### Neonatal MI model.

Cauterization of the LCA was performed on P1 and P3 WT pups of both sexes or on transgenic *αMHC-H2BmCherry* and *CAG-eGFP-anillin* pups (previously published lines made in house) with mixed background (C57BL6J/CD1) (20, 21). Briefly, neonatal mice were anesthetized by hypothermia, followed by lateral thoracotomy at the fourth intercostal space. After exposing the heart surface by removing the pericardium, the left atrium was gently lifted with blunt-end forceps to visualize the root of the LCA, followed by cauterization of the LCA at approximately 1–2 mm distal to the left coronary orifice using a small vessel cauterizer (Fine Science Tools, 18000-03); cauterization was confirmed upon observing the paling of the anterior part of the LV. For chest closure, an 8-0 absorbable suture (Ethicon, V400G) was used; for skin closure, a 10-0 nonabsorbable suture was used (Ethicon, EH7995G). After surgery, the pups were placed on a self-made electric plate set to 37°C until they became responsive and then returned to their mothers. Sham-operated mice underwent the same procedure, including removal of the pericardium, but without cauterization of the LCA. The apex of the LA covering the cauterization site was often slightly damaged, either due to its chafing against the hardened surface of the cauterization scar and/or the lifting of LA by blunt-end forceps despite careful manipulation. For harvesting the hearts, mice were euthanized by decapitation in case of neonates and by cervical dislocation of adults at the indicated time points. Then, heart weight to tibia length and heart weight to body weight ratios were measured. Most of the surviving animals of the P3 MI group had to be sacrificed at 7 dps to comply with German Animal Welfare guidelines, as the mice were unwell and presented signs of heart failure (Figure 1H).

### Histology and immunofluorescent staining.

For tissue preparation, dissected hearts were fixed for 1 hour at 4°C in 4% paraformaldehyde dissolved in PBS solution (4% PFA), and then equilibrated in 30% sucrose-PBS solution overnight at 4°C. For cryosectioning, fixed hearts were embedded in Tissue-Tek O.C.T. compound (Sakura, 4583) and cut into 10-μm-thick sections with a cryostat (Leica CM3050). Sirius red staining with Fast Green (Sigma-Aldrich, F7258) counterstaining was performed to determine the scar size in the heart after surgery. Heart sections were rehydrated in PBS and fixed in Bouin’s Solution (Sigma-Aldrich, HT101128) at 55°C for 1 hour. After washing 3 times with water for 10 minutes each, heart sections were incubated in 0.1% Fast Green for 10 minutes, fixed in 1% acetic acid for 2 minutes, and then incubated in 0.1% Direct Red 80 (Sigma-Aldrich, 365548) for 30 minutes. After washing 3 times with water for 5 minutes each, slides were dehydrated by passing through 3 changes of isopropanol, and then rinsed in 3 changes of xylene and mounted with Entellan (Merck, HX90554761).

For immunostaining, heart sections were fixed for 10 minutes in 4% PFA. An optional step of heat-induced epitope retrieval was performed for the optimization of anti-CDKN1A immunostaining in a microwave tissue processor (Milestone, KOS). After washing 3 times with PBS for 5 minutes each, nonspecific antigens were blocked for 30 minutes in PBS containing 0.2% Triton X-100 and 5% normal donkey serum. Primary antibodies against ACTN2 (clone EA-53) (1:200; Sigma-Aldrich, A7811), aurora kinase B (AURKB) (clone 6) (1:200; BD Biosciences, 611082), cCASP3 (clone Asp175) (1:50; Cell Signaling Technology, 9661L), PTPRC (clone IBL-5/25) (1:1000; Millipore, CBL1326), GFP (1:400; Abcam, ab6662), MKI67 (clone SP6) (1:200; Thermo Fisher Scientific, RM-9106), CDKN1A (clone EPR3993) (1:100; Abcam, ab109199), PECAM1 (clone MEC 13.3) (1:500; BD Pharmingen, 550274), and TNNI3 (1:200; Abcam, ab56357) were diluted in PBS containing 0.2% Triton X-100 and 2.5% normal donkey serum and applied to the sections followed by an overnight incubation at 4°C. After washing 3 times with PBS for 5 minutes each, secondary antibodies conjugated to Cy2, Alexa Fluor 488, Cy3, or Alexa Fluor 647 (all 1:400; Jackson ImmunoResearch) and 0.5 μg/mL DAPI (Invitrogen, R37606), were diluted in PBS, applied to the sections, and incubated for 30 minutes. For some antibodies (anti-PECAM1 and anti-CDKN1A), peroxidase-conjugated secondary antibodies (Vector Labs, Immpress Polymer Reagent Kit) and a tyramide signal amplification kit (PerkinElmer, NEL741001KT, NEL744001KT, and NEL745001KT) were used following the manufacturers’ instructions. After washing 3 times with PBS for 5 minutes each, slides were mounted with Aqua-Poly/Mount (Polysciences, 18606).

Images were acquired using a Zeiss fluorescence microscope (Axion Observer Z.1) with ZEN image analysis software (version 3.1), a Zeiss macroscope (Axio Zoom V.16) with the same software (version 3.1), or a Nikon confocal microscope (Eclipse Ti) with NIS-Elements AR image analysis software (version 4.13).

Injury and scar sizes were determined using TNNI3 staining at 2 dps and Sirius red and Fast Green staining at later stages. A sufficiently large sample size for quantitative measurements was achieved by analyzing 7 sections across the entire heart at approximately equal intervals, as described previously (43). The size of the lesion was calculated as a percentage by normalizing lesion areas to the total LV area from all sections and multiplying the number times 100. RV thickness at 4 dps was determined by measuring the average transmural width of the anterior, middle, and posterior areas of the RV free wall in 7 sections taken from the entire heart at approximately equal intervals.

Cardiac hypertrophy after MI was determined by measuring cross-sectional size of CMs after staining with wheat germ agglutinin (WGA). This was done as for immunostaining, except for an additional 1-hour incubation in fluorescein-labeled WGA (Vector Labs, FL1021; 1:1000 dilution in PBS) before mounting. To rule out technical issues, only CMs with nuclei and symmetrical cross-sectional shapes in the images were included for quantification. To assess the type of CM hypertrophy in the RV, the CM length/width ratio was also measured in the longitudinal sections.

Cell cycle activity was determined by MKI67 staining and analysis of the *CAG-eGFP-anillin* reporter mouse line; for quantitation, the free RV and LV walls were analyzed separately (20). MKI67 staining was used to mark all nonquiescent cells. Nuclear eGFP signals from heart sections of the *CAG-eGFP-anillin* mouse line were used to determine late-interphase cells (19). Cell cycle activity in CMs was quantified as the number of MKI67^+^, or nuclear eGFP^+^, and TNNI3^+^ nuclei on 1 entire section (RV and LV walls were analyzed separately), whereas in non-CMs the number of MKI67^+^TNNI3^–^ nuclei were quantified. To visualize the distribution pattern of cycling CMs, positive CMs in mosaic pictures of whole-heart sections were manually marked with green dots using CorelDRAW (version 22.1) software. In the *CAG-eGFP-anillin* mouse model, eGFP-ANILLIN^+^TNNI3^+^AURKB^+^ costaining was performed to discriminate between cytokinetic and binucleating CMs by determining midbody position and distance between daughter nuclei (19). In this method, CMs undergoing cytokinesis display symmetrical midbody positioning and there is more than 5 μm between 2 daughter nuclei. In the case of binucleation, however, midbody positioning is asymmetric and the distance between daughter nuclei is less than 5 μm. Costaining for AURKB and eGFP excluded staining artifacts and only double-positive cells were included in the analysis; it is possible that CM proliferation was underestimated because of these rigorous criteria. Due to the relatively low number of midbodies per section, 3 to 6 sections per heart were quantified. All numbers of cell cycle activity and of proliferating or binucleating CMs were normalized to the total area of searched sections (free wall of the RV and the LV, separately) (number of cells per mm^2^).

The immune response was assessed by PTPRC staining, and apoptosis by staining against cCASP3 or TUNEL assay using an In Situ Cell Death Detection Kit (Roche, 11684795910) following the manufacturer’s instructions. Due to the fragmentation of apoptotic blebs, only DAPI^+^ signals were included in the quantitation. The number of PTPRC^+^ and apoptotic cells was normalized to the area of the injury (number of cells per mm^2^).

All measurements for size, number, and length in microscopic images were performed using ImageJ software (version 1.48 to 1.52, NIH), Nikon NIS-Elements AR (version 4.13), or Zeiss ZEN microscopy software (version 3.1), respectively.

### Quantification of the percentage of binucleated CMs after cell isolation.

*αMHC-H2Bmcherry* transgenic mice underwent either LCA cauterization or sham procedures, sacrificed, and the hearts harvested on P5. Isolated CMs were obtained using the Neonatal Heart Dissociation Kit (Miltenyi Biotec, 130-098-373) following the manufacturer’s instruction, with the following minor modifications: Instead of the gentleMACs Dissociator, small scissors were used to cut the heart into small tissue pieces. After dissociation, the cell suspension was added to a 24-well glass-bottom plate (Cellvis, P24-1.5H-N) and sedimented to the bottom of the plate by centrifugation (1000*g* for 30 seconds). Images were acquired immediately afterwards using a Nikon confocal microscope (Eclipse Ti).

### Vessel casting.

The 3D structure of coronary arteries was visualized using vessel casting at 21 dps in P1 MI and sham hearts; this was the earliest feasible time point. After harvesting, the aortas were cannulated and perfused with 1 mL PBS (containing 50 IU/mL heparin sodium) to remove excess blood. Microfil compounds (Flow Tech Inc., MV-120 or MV-122) were prepared according to the manufacturer’s instructions and injected into the cannulated ascending aorta. Due to the relatively high viscosity of the Microfil compounds (25 centipoise), only large arteries, but not small vessels, could be filled with the compound. After 20 minutes, hearts were fixed overnight with 4% PFA and then opacified by incubation in glycerol for 2 weeks.

### Echocardiography.

Cardiac function was assessed with echocardiography using a Vevo 3100 equipped with Mx700 (from 1–14 dps) and Mx550 (from 21–120 dps) transducers. The P2–P7 mice were fixed to the heating plate with adhesive tapes on their palms and chins without anesthesia; older mice were put under anesthesia (2%–3% isoflurane with oxygen flow at 1 L/min) and fixed with adhesive tape. Imaging was performed by capturing long- and short-axis views of the hearts always at the middle part of the heart, which was standardized by the hallmark of papillary muscle or aortic valve visualization in the short axis or the long axis view, respectively. The images were captured at 1, 7, 14, 21, and 120 dps for P1 MI and at 1 and 7 dps for P3 MI as well as respective sham mice.

LV function and parameters such as EF, FS, LVEDA, and CO were determined based on long-axis-view images, and LV wall thickness in the short-axis view using the integrated measurement tools of Vevo Lab 3.2.6 software. RVEDA and RVFAC were measured in the short-axis view and are given as percentages using 100 × (RVEDA – RVESA)/RVEDA. RV length was measured in end-diastole in the long-axis view at the middle area of the heart. 4D imaging of the heart was done by scanning the heart using built-in motor and the RV and LV areas were contoured in 3 heart cycles using Vevo Lab software 5.7.1. As an indirect measure of pulmonary arterial pressure, pulsed-wave Doppler of the main pulmonary artery was performed using the same echocardiographic device with the Mx700 transducer. Correct location of the sampling gate was assured in color Doppler imaging. PAAT was measured as the time from the onset of systolic pulmonary blood flow to the peak flow velocity, and PAET as the total time of systolic pulmonary blood flow (23, 24). All echocardiographic data are summarized in Supplemental Table 1.

### RNA-seq and bioinformatics.

For RNA-seq experiments, at 1 dps P1 MI or P3 MI hearts were harvested as well as respective shams. This time point was chosen because earlier work reported maximal changes in gene expression occurring shortly after the lesion (29). Each heart was dissected into the LV free wall, the septum, and RV free wall and snap frozen in liquid nitrogen. DNA-free total RNA was isolated for each compartment of the heart from 4 mice after P1 sham, 6 after P1 MI, 4 after P3 sham, and 9 after P3 MI using the RNeasy Kit (Qiagen) including on-column DNase digestion. RNA quality was controlled by an Agilent Bioanalyzer. For library preparation, the Trio RNA-Seq Library Preparation kit for mouse (TECAN) was used, starting with 50 ng of total RNA. Thirteen PCR cycles were used for library amplification and libraries with an average fragment size of 380 bp were sequenced on a NextSeq 500 in paired-end mode (75 bp, Illumina). Quality control of RNA-seq probes showed issues with a few probes, which were excluded (1 sample from P1 MI, P3 sham, and 2 samples from P3 MI hearts).

For bioinformatic analysis, we used the Galaxy platform (Freiburg Galaxy Project) (44). RNA-seq reads were mapped using RNA STAR (45) followed by counting reads per gene by using FeatureCounts (46). Differentially expressed genes were identified by DESeq2 (47). Data mining for differentially expressed genes with greater than 1.5-fold change and a *P* value of less than 0.05 (adjusted by false discovery rate) was performed using the KNIME Analytics platform (version 4.1). For data visualization, normalization, and cluster analysis, Heatmapper Expression (48) and BioVenn (49) were used. Gene ontology (GO) analysis was performed by DAVID (50) using the KEGG pathway and GO-term databases, with a significance interval for pathways of *P* less than 0.05. *P* values were corrected for multiple testing by the Bonferroni’s step-down method. For all analyses except in Supplemental Figure 5, upregulated and downregulated genes are defined based on comparisons of the MI group with the respective age-matched sham group; in Supplemental Figure 5, upregulated genes are defined based on comparisons of the genes in the LV versus the RV (Supplemental Figure 5I) or RV versus LV (Supplemental Figure 5J) in the same heart.

### Statistics.

Data were analyzed by Student’s *t* test or 1-way ANOVA using GraphPad Prism (version 9.41), as indicated in the figure legends. All data are presented as mean ± SEM, and *n* values indicate the number of individual animals. Significance was accepted when *P* was less than 0.05. All statistically analyzed figures were produced by GraphPad Prism (version 9.41). All quantitative morphological and cell biological data are listed in Supplemental Table 2.

### Study approval.

All animal procedures were conducted in accordance with the guidelines from Directive 2010/63/EU of the European Parliament on the protection of animals used for scientific purposes and were approved by the local ethics review board (84-02.04.2014.A161 and 81-02.04.2020.A463 Landesamt für Natur, Umwelt und Verbraucherschutz, Nordrhein-Westfalen, Germany).

### Data availability.

All RNA-seq data sets reported in this manuscript are deposited in the Short Read Archive at the NCBI under BioProject ID PRJNA941198. Data associated with the main and supplemental figures are provided in the Supporting Data Values Excel file.

## Author contributions

TH established the surgical method and performed the cell biological and histological analyses. MMM analyzed the RNA-seq data and performed echocardiography. TH and MMM assembled and compiled the figures. FE measured and analyzed pulmonary arterial blood flow parameters. MH contributed to the establishment of the neonatal lesion model and planned and provided input for the cell biological analyses and the RNA-seq data. MH, MIK, and BKF designed the experiments. MH and BKF supervised the experimental work. All authors discussed the results and commented on the manuscript. MIK and BKF wrote the manuscript.

## Supplementary Material

Supplemental data

Supplemental videos 1-6

Supporting data values

## Version 1 02/06/2024

In-Press Preview

## Version 2 03/08/2024

Electronic publication
