# Supplementary material for: Right ventricular cardiomyocyte expansion accompanies cardiac regeneration in newborn mice after large left ventricular infarcts
Source: JCI Insight. 2024 Feb 6;9(5):e176281. doi: 10.1172/jci.insight.176281 (PMC11143925; doi:10.1172/jci.insight.176281)
Supplement: Supplemental data [file jciinsight-9-176281-s056.pdf]

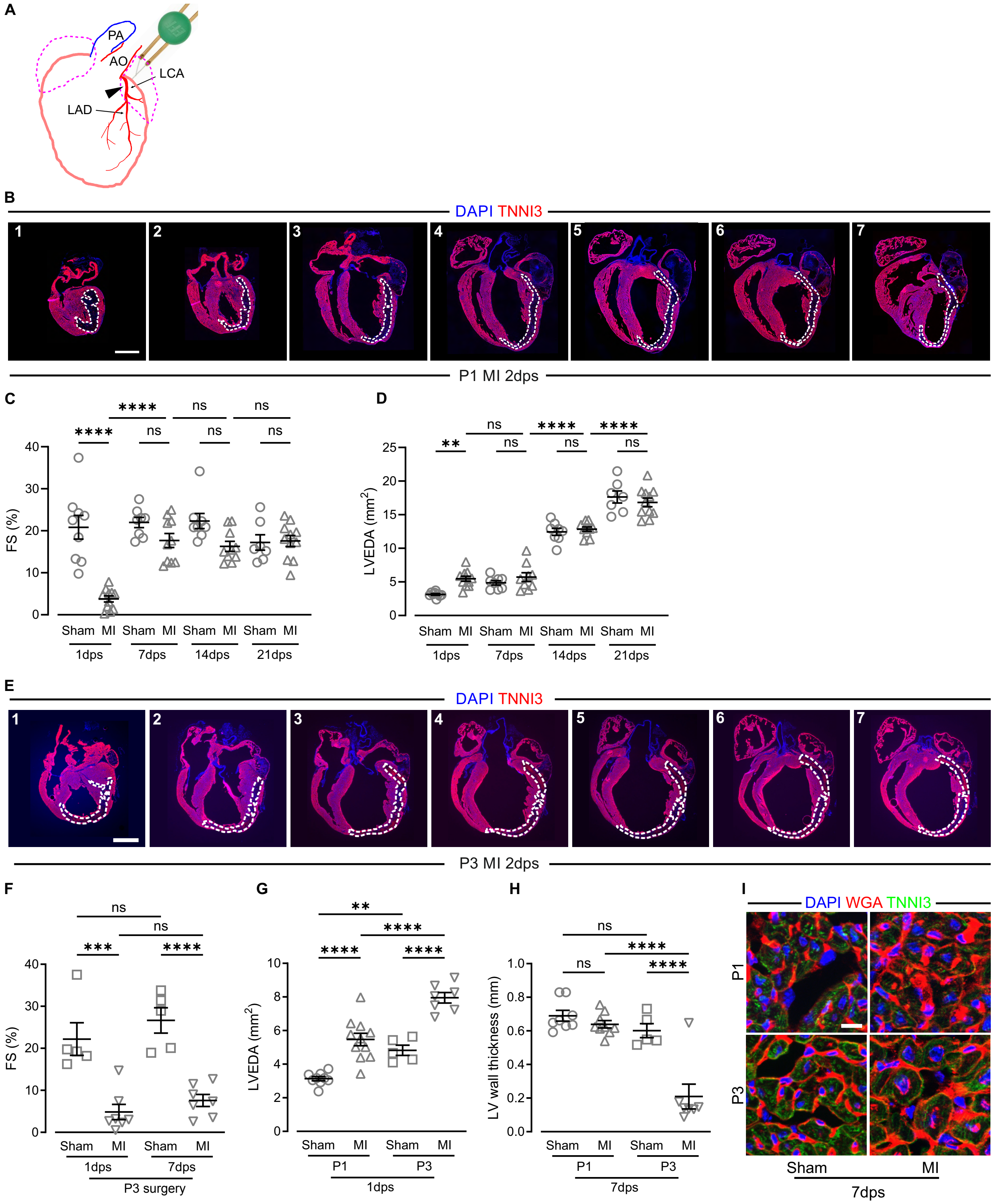
A
PA
AO
LCA
LAD
B
DAPI TNNI3
1
2
3
4
5
6
7
P1 MI 2dps
C
****
ns
ns
****
ns
ns
ns
FS (%)
40
30
20
10
0
Sham MI Sham MI Sham MI Sham MI
1dps 7dps 14dps 21dps
D
ns
****
****
**
ns
ns
ns
LVEDA (mm2)
25
20
15
10
5
0
Sham MI Sham MI Sham MI Sham MI
1dps 7dps 14dps 21dps
E
DAPI TNNI3
1
2
3
4
5
6
7
P3 MI 2dps
F
ns
ns
***
****
FS (%)
40
30
20
10
0
Sham MI Sham MI
1dps 7dps
P3 surgery
G
**
****
****
****
LVEDA (mm2)
10
8
6
4
2
0
Sham MI Sham MI
P1 P3
1dps
H
ns
****
ns
****
LV wall thickness (mm)
1.0
0.8
0.6
0.4
0.2
0.0
Sham MI Sham MI
P1 P3
7dps
I
DAPI WGA TNNI3
P1
P3
Sham MI
7dps

**Supplemental Figure 1: Histological and echocardiographic analysis of P1 and P3 MI, and sham hearts.**

(**A**) Scheme of the cauterization injury of the LCA (black arrowhead) in the neonatal mouse heart; PA = Pulmonary Artery; Ao = Aorta; LAD = Left Anterior Descending coronary artery; LCA = Left Coronary Artery. (**B**) Serial sections taken at approximately equal distance through a P1 MI heart co-stained against TNNI3 and DAPI; dashed lines mark infarct areas; scale bar=1mm. (**C** and **D**) Echocardiographic assessment of fractional shortening (FS) (C) and LV end-diastolic area (LVEDA) (D) after P1 surgery. (**E**) Serial sections through a P3 MI heart co-stained as in A; scale bar=1mm. (**F-H**) Echocardiographic assessment of FS (F), LVEDA (G) and LV wall thickness (H). (**I**) CM cross-sectional area measured in heart sections co-stained against WGA, TNNI3 and DAPI. Scale bar=20µm; (**p<0.01; ****p<0.0001; ns = no significance; one-way ANOVA with Holm-Šídák post hoc test).

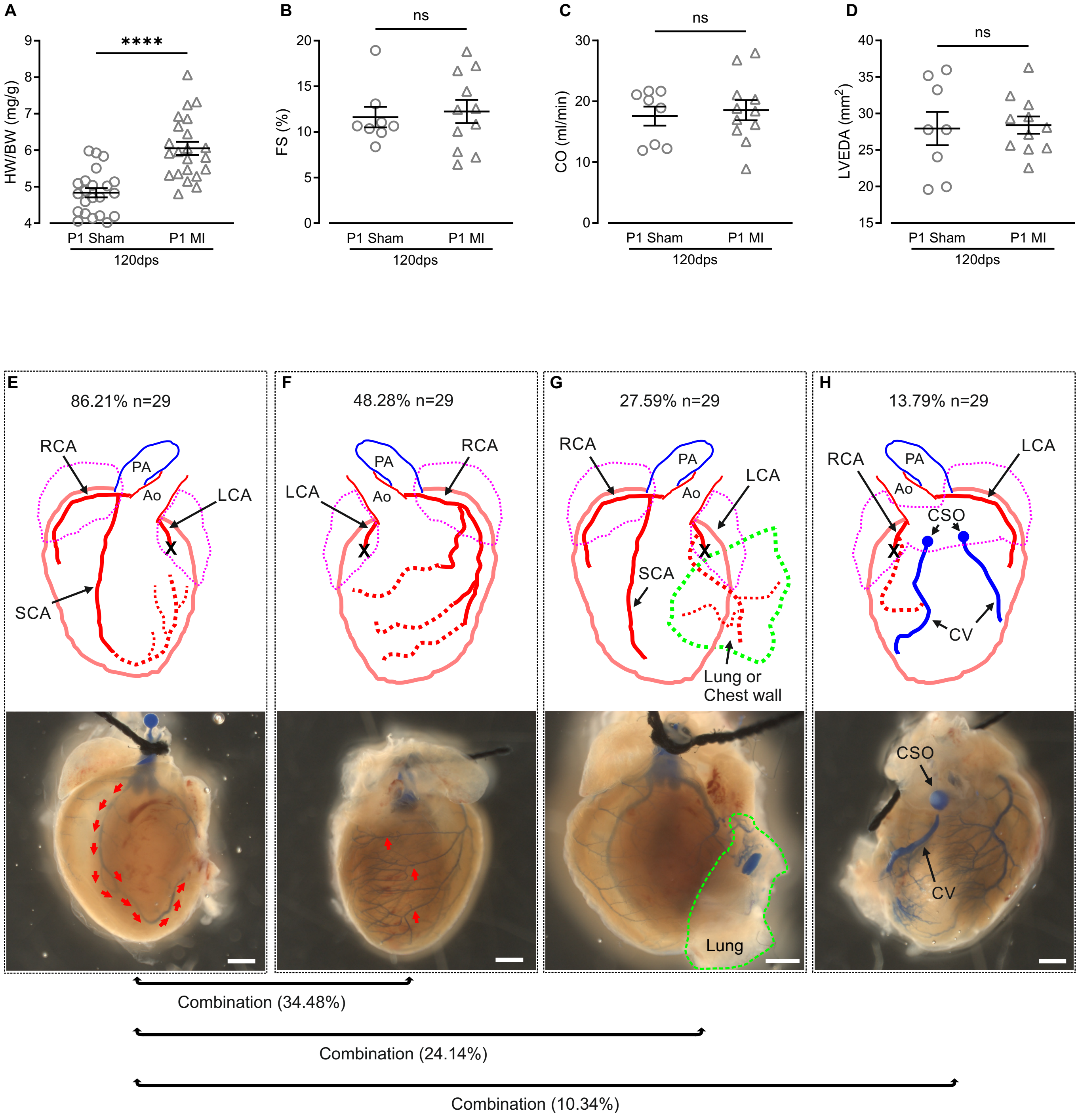

A
HW/BW (mg/g)
****
P1 Sham
P1 MI
120dps
B
FS (%)
ns
P1 Sham
P1 MI
120dps
C
CO (ml/min)
ns
P1 Sham
P1 MI
120dps
D
LVEDA (mm2)
ns
P1 Sham
P1 MI
120dps
E
86.21% n=29
RCA
PA
Ao
LCA
SCA
F
48.28% n=29
LCA
PA
Ao
RCA
G
27.59% n=29
RCA
PA
Ao
LCA
SCA
Lung or
Chest wall
Lung
H
13.79% n=29
RCA
PA
Ao
LCA
CSO
CV
Combination (34.48%)
Combination (24.14%)
Combination (10.34%)

**Supplemental Figure 2: Histological and echocardiographic analysis of P1 MI and sham hearts at 120 dps, vessel casting in P1 MI hearts at 21 dps.**

(**A**) Quantitation of heart- to body weight (HW/BW) ratio. (**B-D**) Echocardiographic assessment of FS (B), Cardiac Output (CO) (C) and LVEDA (D). (**E-H**) Schematics (upper panels) of collateral vessels connecting the LV coronary artery to septal coronary artery (E), right coronary artery (F), lung or other extra cardiac vessels (G) and coronary veins (H). Images of coronary artery vessel casting (lower panels) in P1 MI hearts; red arrows mark the extension of branches of septal (E) and right coronary artery (F), numbers indicate frequencies of the different variations. Scale bar=1mm. (CV = Coronary Vein; CSO = Coronary Sinus Orifice; ****$p<0.0001$; ns = no significance, unpaired t-test).

**A**

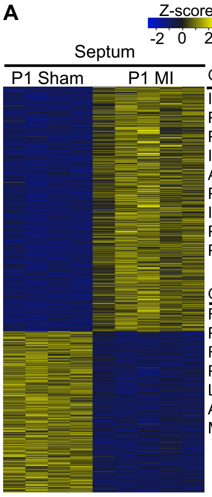


| Gene ontology of upregulated genes | # of Genes | -log (P-value) |
|---|---|---|
| Inflammatory response | 44 | 14.49321 |
| Positive regulation of cell migration | 31 | 12.15224 |
| Positive regulation of inflammatory response | 18 | 11.4507 |
| Immune system process | 38 | 9.228095 |
| Angiogenesis | 27 | 7.697353 |
| Positive regulation of cell proliferation | 41 | 6.624964 |
| Innate immune response | 33 | 6.162225 |
| Positive regulation of angiogenesis | 16 | 5.392967 |
| Positive regulation of ERK1 and ERK2 cascade | 20 | 5.311235 |

| Gene ontology of downregulated genes | # of Genes | -log (P-value) |
|---|---|---|
| Fatty acid beta-oxidation | 5 | 2.043763 |
| Positive regulation of fatty acid oxidation | 3 | 2.043125 |
| Fatty acid metabolic process | 9 | 2.038974 |
| Positive regulation of fatty acid beta-oxidation | 3 | 1.939344 |
| Lipid metabolic process | 17 | 1.887173 |
| Angiogenesis | 11 | 1.82281 |
| Metabolic process | 15 | 1.268561 |

**B**

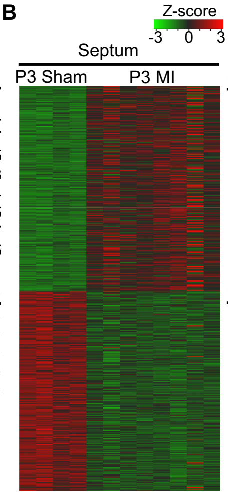


| Gene ontology of upregulated genes | # of Genes | -log (P-value) |
|---|---|---|
| Inflammatory response | 24 | 5.598298 |
| Positive regulation of cell migration | 20 | 6.903576 |
| Positive regulation of inflammatory response | 11 | 5.967721 |
| Angiogenesis | 29 | 12.26002 |
| Positive regulation of cell proliferation | 34 | 6.839406 |
| Positive regulation of angiogenesis | 13 | 4.82976 |
| Positive regulation of ERK1 and ERK2 cascade | 15 | 4.12601 |

| Gene ontology of downregulated genes | # of Genes | -log (P-value) |
|---|---|---|
| Cell cycle | 35 | 7.217229 |
| DNA replication | 15 | 6.849056 |
| Cell division | 20 | 3.773737 |
| Metabolic process | 22 | 3.440299 |
| DNA replication initiation | 5 | 2.93956 |
| Mitotic nuclear division | 13 | 2.040041 |
| Positive regulation of fatty acid oxidation | 3 | 1.99973 |
| Negative regulation of cell growth | 8 | 1.923943 |
| Fatty acid metabolic process | 9 | 1.912661 |
| Oxidation-reduction process | 23 | 1.826911 |
| Regulation of mitotic cell cycle | 4 | 1.444837 |

**C**

| GO (Unique P1 upregulated genes) | #Gene |
|---|---|
| Immune system process | 29 |
| Inflammatory response | 28 |
| Innate immune response | 24 |
| Positive regulation of cell proliferation | 19 |
| Negative regulation of apoptotic process | 19 |
| Positive regulation of cell migration | 18 |
| Positive regulation of apoptotic process | 18 |
| Immune response | 15 |
| Regulation of cell proliferation | 14 |
| Regulation of apoptotic process | 13 |
| Positive regulation of ERK1 and ERK2 cascade | 10 |
| Cell proliferation | 10 |

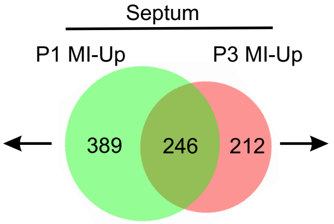


| GO (Unique P3 upregulated genes) | #Gene |
|---|---|
| Positive regulation of cell proliferation | 12 |
| Angiogenesis | 11 |
| Negative regulation of cell proliferation | 11 |
| Negative regulation of apoptotic process | 11 |
| Response to hypoxia | 10 |
| Inflammatory response | 8 |
| Wnt signaling pathway | 7 |

**Supplemental Figure 3: Transcriptome analysis of the septum in P1 and P3 MI and sham hearts at 1 dps.**

(**A** and **B**) Heatmap showing differentially up- (upper part of tables) and downregulated (lower part of tables) genes and selected GOs of biological processes in the septum of P1 (A) and P3 MI (B) hearts compared to sham hearts. (**C**) Venn diagram illustrating overlap of overexpressed genes in the septum. Left (P1) and right (P3) tables display selected GOs of biological processes of uniquely upregulated genes in MI hearts.

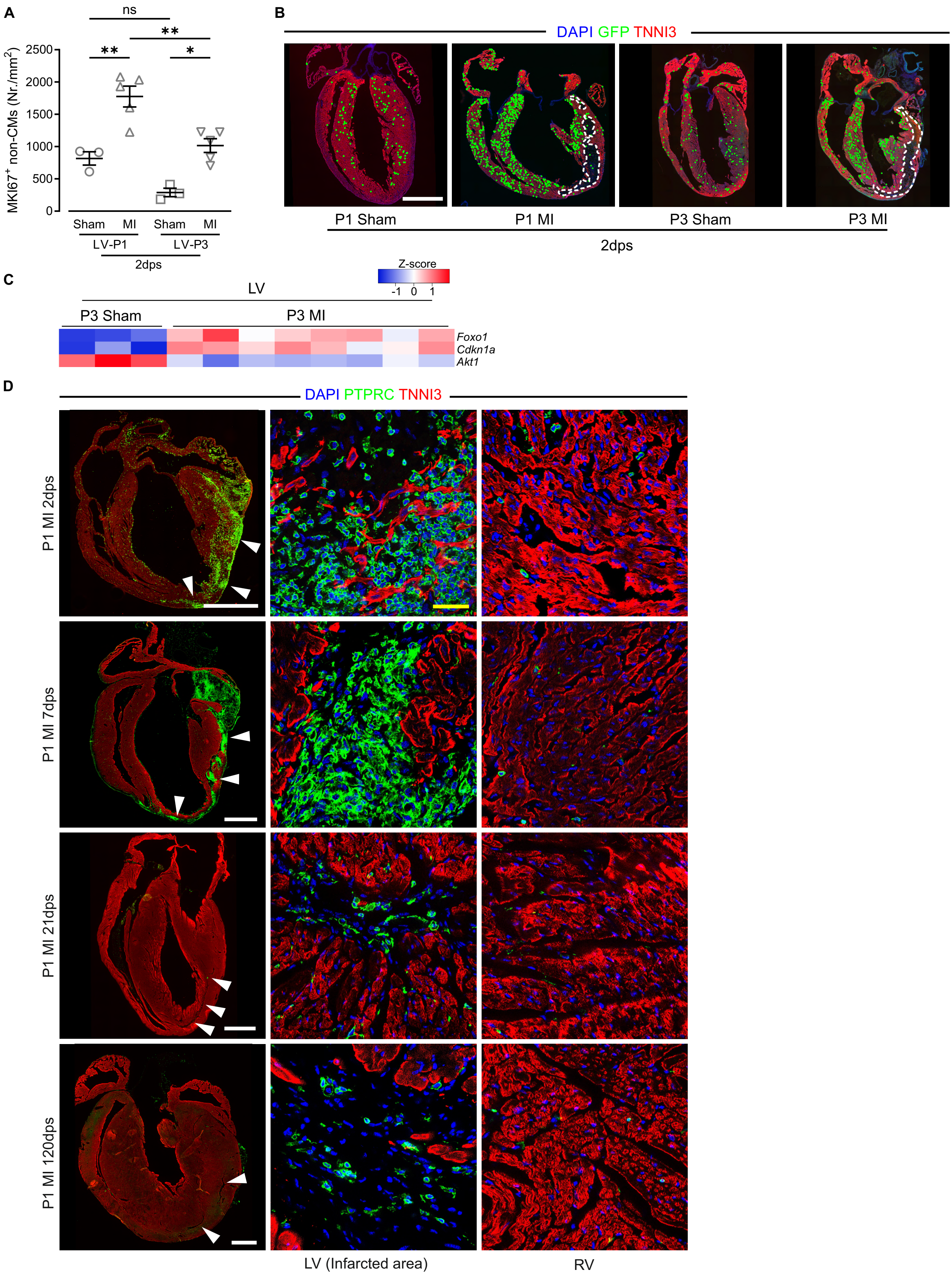
A
ns
**
**
*
MKI67+ non-CMs (Nr./mm2)
2500
2000
1500
1000
500
0
Sham
MI
Sham
MI
LV-P1
LV-P3
2dps
B
DAPI GFP TNNI3
P1 Sham
P1 MI
P3 Sham
P3 MI
2dps
C
Z-score
-1
0
1
LV
P3 Sham
P3 MI
Foxo1
Cdkn1a
Akt1
D
DAPI PTPRC TNNI3
P1 MI 2dps
P1 MI 7dps
P1 MI 21dps
P1 MI 120dps
LV (Infarcted area)
RV

**Supplemental Figure 4: Increased proliferation of non-CMs and CMs, and strong inflammation in P1 and P3 MI, and sham hearts.**

(**A**) Quantitation of MKI67+ non-CMs in LV heart sections. (**B**) Mosaic whole-heart images of *CAG-eGFP-anillin* hearts co-stained against eGFP and TNNI3. eGFP+ CMs are manually marked with green dots to visualize the distribution pattern of cycling CMs. (**C**) Heatmap showing the gene expression of *Foxo1, Cdkn1a* and *Akt1* in the LV of MI and sham hearts. (**D**) Mosaic (left panels) and magnified images (middle and right panels) of heart sections co-stained against PTPRC and TNNI3; arrowheads indicate infarcted or scarred areas; white scale bars=1mm; yellow scale bar=40µm. (*p<0.05; **p<0. 01; ns = no significance; one-way ANOVA with Holm-Šídák post hoc test).

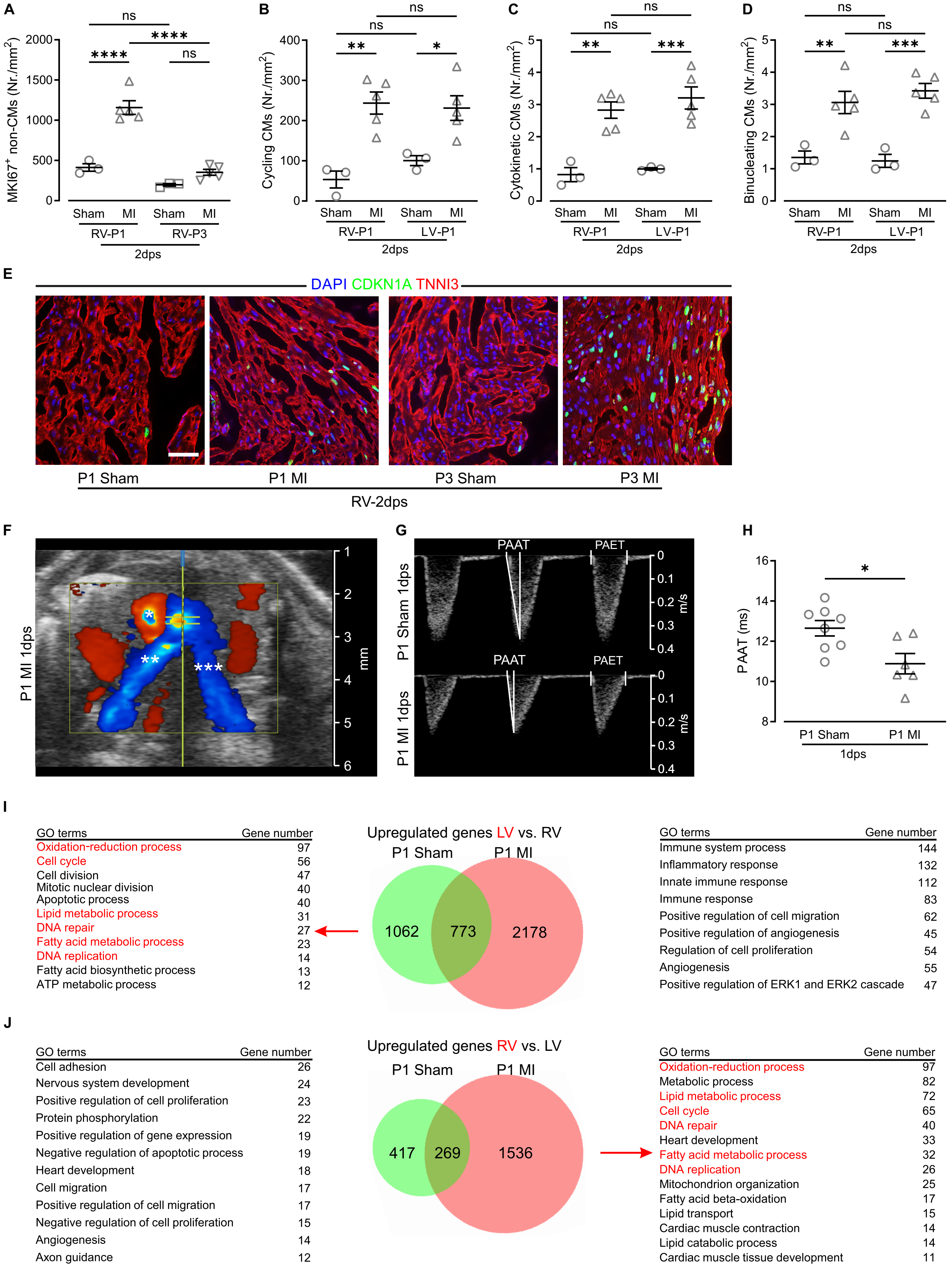
A
MKI67+ non-CMs (Nr./mm2)
ns
****
****
ns
Sham MI Sham MI
RV-P1 RV-P3
2dps
B
Cycling CMs (Nr./mm2)
ns
ns
**
*
Sham MI Sham MI
RV-P1 LV-P1
2dps
C
Cytokinetic CMs (Nr./mm2)
ns
ns
**
***
Sham MI Sham MI
RV-P1 LV-P1
2dps
D
Binucleating CMs (Nr./mm2)
ns
ns
**
***
Sham MI Sham MI
RV-P1 LV-P1
2dps
E
DAPI CDKN1A TNNI3
P1 Sham
P1 MI
P3 Sham
P3 MI
RV-2dps
F
P1 MI 1dps
mm
G
PAAT
PAET
P1 Sham 1dps
P1 MI 1dps
m/s
H
PAAT (ms)
*
P1 Sham P1 MI
1dps
I
GO terms Gene number
Oxidation-reduction process 97
Cell cycle 56
Cell division 47
Mitotic nuclear division 40
Apoptotic process 40
Lipid metabolic process 31
DNA repair 27
Fatty acid metabolic process 23
DNA replication 14
Fatty acid biosynthetic process 13
ATP metabolic process 12
Upregulated genes LV vs. RV
P1 Sham P1 MI
1062 773 2178
GO terms Gene number
Immune system process 144
Inflammatory response 132
Innate immune response 112
Immune response 83
Positive regulation of cell migration 62
Positive regulation of angiogenesis 45
Regulation of cell proliferation 54
Angiogenesis 55
Positive regulation of ERK1 and ERK2 cascade 47
J
GO terms Gene number
Cell adhesion 26
Nervous system development 24
Positive regulation of cell proliferation 23
Protein phosphorylation 22
Positive regulation of gene expression 19
Negative regulation of apoptotic process 19
Heart development 18
Cell migration 17
Positive regulation of cell migration 17
Negative regulation of cell proliferation 15
Angiogenesis 14
Axon guidance 12
Upregulated genes RV vs. LV
P1 Sham P1 MI
417 269 1536
GO terms Gene number
Oxidation-reduction process 97
Metabolic process 82
Lipid metabolic process 72
Cell cycle 65
DNA repair 40
Heart development 33
Fatty acid metabolic process 32
DNA replication 26
Mitochondrion organization 25
Fatty acid beta-oxidation 17
Lipid transport 15
Cardiac muscle contraction 14
Lipid catabolic process 14
Cardiac muscle tissue development 11

**Supplemental Figure 5: Proliferation, CDKN1A protein expression, and transcriptome analysis (at 1 dps) in the RV and LV of P1, P3 MI and sham hearts; Doppler measurements of the blood flow in the pulmonary artery in P1 MI and sham hearts at 1 dps.**

(**A**) Quantitation of MKI67$^{+}$ non-CMs in RV. (**B-D**) Quantitation of cycling (B), cytokinetic (C) and binucleating (D) CMs in RV and LV of *CAG-eGFP-anillin* mice. (**E**) RV heart sections co-stained against CDKN1A, TNNI3 and DAPI; scale bar=40µm. (**F**) Colour doppler of the main pulmonary artery in a P1 MI heart: *ascending aorta; **right pulmonary artery; ***left pulmonary artery. (**G**) Flow velocity curve measured in the main pulmonary artery in a P1 MI and sham heart; PAAT: pulmonary artery acceleration time; PAET: pulmonary artery ejection time. (**H**) Doppler measurements of PAAT in the pulmonary artery. (**I** and **J**) Venn diagram comparing upregulated genes in the LV to RV (I) and in the RV to LV (J) in MI and sham hearts. Tables on the left and right show representative GO of biological processes, similar GO terms in sham LV (I) and MI RV (J) hearts are highlighted in red. *p<0.05; **p<0.01; ***p<0.001; ****p<0.0001; ns = no significance; A-D: one-way ANOVA with Holm-Šídák post hoc test; H unpaired t-test).

## Supplemental Table 1: Echocardiography parameters

| Condition | Time point | EF ± SEM (%) | FS ± SEM (%) | CO ± SEM (ml/min) | LVEDA ± SEM ($mm^2$) | Wall thickness ± SEM (mm) | RV FAC ± SEM (%) | RV length ± SEM (mm) | RVEDA ± SEM ($mm^2$) | PAAT ± SEM (ms) | PAAT/PAET ± SEM (ratio) |
|---|---|---|---|---|---|---|---|---|---|---|---|
| **P1 Sham** | 1dps | 86.02 ± 2.39<br>n=9 | 20.82 ± 2.81<br>n=9 | | 3.14 ± 0.13<br>n=9 | 0.40 ± 0.03<br>n=9 | 57.07 ± 6.13<br>n=9 | 4.00 ± 0.06<br>n=9 | 0.77 ± 0.07<br>n=9 | 12.65 ± 0.38<br>n=8 | 0.23 ± 0.01<br>n=8 |
| | 7dps | 79.10 ± 2.02<br>n=8 | 21.97 ± 1.21<br>n=8 | | 4.86 ± 0.33<br>n=8 | 0.69 ± 0.03<br>n=8 | 46.46 ± 3.68<br>n=8 | 6.09 ± 0.31<br>n=7 | 1.57 ± 0.17<br>n=8 | | |
| | 14dps | 70.48 ± 3.65<br>n=8 | 22.31 ± 1.81<br>n=8 | | 12.45 ± 0.52<br>n=8 | | | 7.90 ± 0.30<br>n=7 | | | |
| | 21dps | 67.34 ± 2.41<br>n=7 | 17.22 ± 1.83<br>n=7 | | 17.63 ± 0.90<br>n=7 | | | 9.75 ± 0.15<br>n=7 | | | |
| | 120dps | 43.79 ± 3.34<br>n=8 | 11.62 ± 1.14<br>n=8 | 17.58 ± 1.56<br>n=8 | 27.94 ± 2.29<br>n=8 | | | 12.48 ± 0.33<br>n=7 | | | |
| **P1 MI** | 1dps | 15.92 ± 2.87<br>n=11 | 3.78 ± 0.74<br>n=11 | | 5.47 ± 0.32<br>n=11 | 0.31 ± 0.02<br>n=11 | 48.44 ± 5.61<br>n=11 | 4.55 ± 0.11<br>n=11 | 0.70 ± 0.07<br>n=11 | 10.88 ± 0.51<br>n=6 | 0.17 ± 0.01<br>n=6 |
| | 7dps | 60.63 ± 4.37<br>n=10 | 17.67 ± 1.66<br>n=10 | | 5.71 ± 0.66<br>n=9 | 0.64 ± 0.02<br>n=9 | 46.23 ± 3.28<br>n=10 | 7.65 ± 0.24<br>n=11 | 1.50 ± 0.19<br>n=10 | | |
| | 14dps | 58.40 ± 2.14<br>n=10 | 16.29 ± 1.19<br>n=10 | | 12.85 ± 0.33<br>n=10 | | | 11.22 ± 0.25<br>n=11 | | | |
| | 21dps | 59.31 ± 2.23<br>n=11 | 17.57 ± 1.31<br>n=11 | | 16.82 ± 0.64<br>n=11 | | | 13.06 ± 0.31<br>n=11 | | | |
| | 120dps | 39.87 ± 3.80<br>n=11 | 12.24 ± 1.27<br>n=11 | 18.56 ± 1.66<br>n=11 | 28.41 ± 1.18<br>n=11 | | | 16.72 ± 0.37<br>n=11 | | | |
| **P3 Sham** | 1dps | 79.57 ± 2.72<br>n=5 | 22.19 ± 3.88<br>n=5 | | 4.82 ± 0.31<br>n=5 | 0.41 ± 0.02<br>n=5 | 52.66 ± 5.89<br>n=5 | | 0.91 ± 0.15<br>n=5 | | |
| | 7dps | 83.91 ± 3.61<br>n=5 | 26.64 ± 3.01<br>n=5 | | 6.37 ± 0.66<br>n=5 | 0.60 ± 0.04<br>n=5 | 58.55 ± 3.74<br>n=5 | | 2.14 ± 0.43<br>n=5 | | |
| **P3 MI** | 1dps | 8.79 ± 1.00<br>n=7 | 4.83 ± 1.83<br>n=7 | | 7.95 ± 0.31<br>n=7 | 0.23 ± 0.02<br>n=7 | 23.70 ± 4.20<br>n=8 | | 1.72 ± 0.11<br>n=8 | | |
| | 7dps | 10.02 ± 1.07<br>n=7 | 7.59 ± 1.44<br>n=7 | | 16.50 ± 1.73<br>n=7 | 0.21 ± 0.07<br>n=7 | 15.88 ± 3.80<br>n=7 | | 3.43 ± 0.45<br>n=7 | | |

**Supplemental Table 2: Descriptive statistics**

| Parameter | Condition | n= | Average ± S.E.M | Related figures |
|---|---|---|---|---|
| Injury/scar size (%) | P1 MI 2dps | 5 | 38.99 ± 1.18 | Figure 1B |
| | P1 MI 7dps | 5 | 19.40 ± 2.88 | |
| | P1 MI 21dps | 5 | 6.59 ± 0.33 | |
| | P1 MI 120dps | 4 | 2.92 ± 0.10 | |
| | P3 MI 2dps | 6 | 42.74 ± 1.64 | Figure 1E |
| | P3 MI 7dps | 6 | 70.41 ± 2.99 | |
| CM cross-sectional area (μm$^2$) | P1 Sham 7dps LV | 4 | 63.23 ± 3.59 | Figure 1L |
| | P1 MI 7dps LV | 8 | 82.10 ± 3.55 | |
| | P3 Sham 7dps LV | 3 | 63.21 ± 6.01 | |
| | P3 MI 7dps LV | 6 | 110.11 ± 1.06 | |
| | P1 Sham 120dps RV | 7 | 259.45 ± 22.43 | Figure 2N |
| | P1 MI 120dps RV | 8 | 371.70 ± 23.11 | |
| | P3 Sham 120dps LV | 7 | 271.17 ± 9.75 | |
| | P3 MI 120dps LV | 8 | 346.33 ± 23.11 | |
| | P1 Sham 4dps RV | 3 | 34.52 ± 0.24 | Figure 8D |
| | P1 MI 4dps RV | 3 | 39.44 ± 1.04 | |
| | P3 Sham 4dps RV | 3 | 41.37 ± 0.88 | |
| | P3 MI 4dps RV | 3 | 84.88 ± 2.11 | |
| CM length/width (ratio) in RV | P1 Sham 120dps | 7 | 6.55 ± 0.25 | Figure 2J |
| | P1 MI 120dps | 9 | 6.98 ± 0.21 | |
| HW/TL (g/mm) | P1 Sham 120dps | 22 | 0.0093 ± 0.00043 | Figure 2L |
| | P1 MI 120dps | 22 | 0.012 ± 0.00048 | |
| HW/BW (mg/g) | P1 Sham 120dps | 22 | 4.84 ± 0.13 | Supplemental Figure 2A |
| | P1 MI 120dps | 22 | 6.05 ± 0.18 | |
| MKI67$^+$ CMs (Nr./mm$^2$) | P1 Sham 2dps LV | 3 | 329.40 ± 26.65 | Figure 4B Figure 7B-C |
| | P1 MI 2dps LV | 5 | 506.17 ± 31.93 | |
| | P3 Sham 2dps LV | 3 | 162.62 ± 6.55 | |
| | P3 MI 2dps LV | 5 | 274.28 ± 23.12 | |
| | P1 Sham 2dps RV | 3 | 248.79 ± 28.46 | Figure 7B |
| | P1 MI 2dps RV | 5 | 657.32 ± 30.30 | |
| | P3 Sham 2dps RV | 3 | 125.84 ± 22.00 | Figure 7C |
| | P3 MI 2dps RV | 5 | 233.24 ± 17.76 | |
| MKI67$^+$ non-CMs (Nr./mm$^2$) | P1 Sham 2dps LV | 3 | 816.47 ± 102.47 | Supplemental Figure 4A |
| | P1 MI 2dps LV | 5 | 1775.05 ± 160.65 | |
| | P3 Sham 2dps LV | 3 | 289.51 ± 66.04 | |
| | P3 MI 2dps LV | 5 | 1015.25 ± 106.29 | |
| | P1 Sham 2dps RV | 3 | 412.32 ± 46.57 | Supplemental Figure 5A |
| | P1 MI 2dps RV | 5 | 1157.28 ± 84.57 | |
| | P3 Sham 2dps RV | 3 | 196.72 ± 15.80 | |
| | P3 MI 2dps RV | 5 | 352.37 ± 36.18 | |

**Supplemental Table 2 (continued)**

| Parameter | Condition | n= | Average ± S.E.M | Related figures |
|---|---|---|---|---|
| Cycling CMs (Nr./mm²) | P1 Sham 2dps LV | 3 | 100.42 ± 12.32 | Figure 4D Supplemental Figure 5B |
| | P1 MI 2dps LV | 5 | 231.04 ± 30.64 | |
| | P3 Sham 2dps LV | 7 | 78.92 ± 17.85 | |
| | P3 MI 2dps LV | 7 | 78.67 ± 21.16 | |
| | P1 Sham 2dps RV | 3 | 53.66 ± 21.00 | Figure 7D Supplemental Figure 5B |
| | P1 MI 2dps RV | 5 | 243.60 ± 27.55 | |
| | P3 Sham 2dps RV | 7 | 41.69 ± 7.88 | |
| | P3 MI 2dps RV | 7 | 132.58 ± 23.45 | |
| Cytokinetic CMs (Nr./mm²) | P1 Sham 2dps LV | 3 | 1.00 ± 0.04 | Figure 4F Supplemental Figure 5C |
| | P1 MI 2dps LV | 5 | 3.20 ± 0.34 | |
| | P3 Sham 2dps LV | 3 | 0.19 ± 0.05 | |
| | P3 MI 2dps LV | 4 | 0.40 ± 0.06 | |
| | P1 Sham 2dps RV | 3 | 0.82 ± 0.22 | Figure 7E Supplemental Figure 5C |
| | P1 MI 2dps RV | 5 | 2.83 ± 0.26 | |
| | P3 Sham 2dps RV | 3 | 0.23 ± 0.02 | |
| | P3 MI 2dps RV | 4 | 0.69 ± 0.11 | |
| Binucleating CMs (Nr./mm²) | P1 Sham 2dps LV | 3 | 1.35 ± 0.20 | Figure 4G Supplemental Figure 5D |
| | P1 MI 2dps LV | 5 | 3.06 ± 0.35 | |
| | P3 Sham 2dps LV | 3 | 0.70 ± 0.03 | |
| | P3 MI 2dps LV | 4 | 1.38 ± 0.28 | |
| | P1 Sham 2dps RV | 3 | 1.25 ± 0.20 | Figure 7F Supplemental Figure 5D |
| | P1 MI 2dps RV | 5 | 3.42 ± 0.23 | |
| | P3 Sham 2dps RV | 3 | 0.68 ± 0.11 | |
| | P3 MI 2dps RV | 4 | 1.51 ± 0.18 | |
| CM binucleation rate at P5 (%) | P1 Sham | 3 | 12.12 ± 0.96 | Figure 4I |
| | P1 MI | 9 | 16.98 ± 0.90 | |
| | P3 Sham | 6 | 10.51 ± 0.54 | |
| | P3 MI | 6 | 12.47 ± 0.84 | |
| $CDKN1A^+$ CMs (Nr./mm²) | P1 Sham 2dps LV | 4 | 102.97 ± 37.79 | Figure 5B |
| | P1 MI 2dps LV | 6 | 322.90 ± 46.25 | |
| | P3 Sham 2dps LV | 6 | 94.73 ± 13.68 | |
| | P3 MI 2dps LV | 5 | 788.32 ± 160.20 | |
| | P1 Sham 2dps RV | 4 | 76.68 ± 16.73 | Figure 7G |
| | P1 MI 2dps RV | 6 | 345.39 ± 15.98 | |
| | P3 Sham 2dps RV | 6 | 79.23 ± 8.83 | |
| | P3 MI 2dps RV | 5 | 709.35 ± 86.90 | |

**Supplemental Table 2 (continued)**

| Parameter | Condition | n= | Average ± S.E.M | Related figures |
|---|---|---|---|---|
| cCASP3+/DAPI+ CMs (Nr./mm$^2$) | P1 Sham 2dps | 3 | 512.95 ± 111.00 | Figure 5E |
| | P3 MI 2dps | 3 | 1208.80 ± 85.06 | |
| TUNEL+/DAPI+ CMs (Nr./mm$^2$) | P1 Sham 2dps | 5 | 235.37 ± 30.05 | Figure 5G |
| | P3 MI 2dps | 5 | 1252.18 ± 165.46 | |
| PTPRC+/DAPI+ CMs (Nr./mm$^2$) | P1 Sham 2dps | 5 | 2190.26 ± 397.03 | Figure 5I |
| | P3 MI 2dps | 5 | 509.07 ± 44.28 | |
| Capillary density (EC/CM) | P1 Sham 7dps LV | 4 | 0.92 ± 0.03 | Figure 5K |
| | P1 MI 7dps LV | 8 | 0.96 ± 0.04 | |
| | P3 Sham 7dps LV | 3 | 1.16 ± 0.04 | |
| | P3 MI 7dps LV | 6 | 0.91 ± 0.05 | |
| | P1 Sham 7dps RV | 4 | 0.92 ± 0.05 | Figure 8F |
| | P1 MI 7dps RV | 8 | 1.16 ± 0.03 | |
| | P3 Sham 7dps RV | 3 | 1.00 ± 0.07 | |
| | P3 MI 7dps RV | 6 | 0.80 ± 0.02 | |
| RV thickness (µm) | P1 Sham 4dps | 3 | 256.40 ± 4.43 | Figure 8B |
| | P1 MI 4dps | 3 | 369.36 ± 20.79 | |
| | P3 Sham 4dps | 3 | 403.40 ± 3.23 | |
| | P3 MI 4dps | 3 | 324.14 ± 9.83 | |
