# Supplementary material for: Right ventricular cardiomyocyte expansion accompanies cardiac regeneration in newborn mice after large left ventricular infarcts
Source: JCI Insight. 2024 Feb 6;9(5):e176281. doi: 10.1172/jci.insight.176281 (PMC11143925; doi:10.1172/jci.insight.176281)
Supplement: Supplemental videos 1-6 [file jciinsight-9-176281-s057.pptx]

## Slide 1
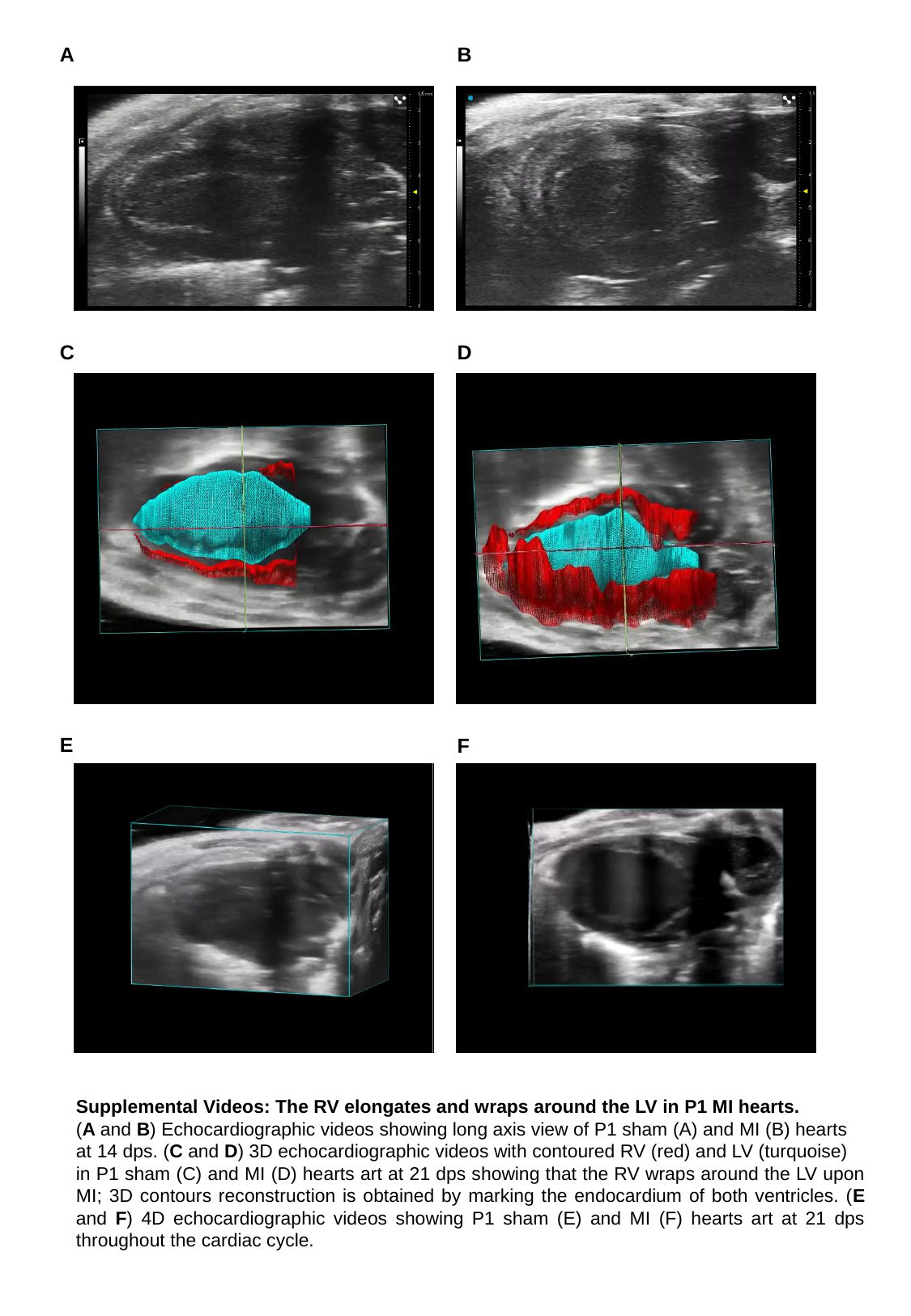

A
B
C
D
E
F
Supplemental Videos: The RV elongates and wraps around the LV in P1 MI hearts.
(A and B) Echocardiographic videos showing long axis view of P1 sham (A) and MI (B) hearts
at 14 dps. (C and D) 3D echocardiographic videos with contoured RV (red) and LV (turquoise)
in P1 sham (C) and MI (D) hearts art at 21 dps showing that the RV wraps around the LV upon MI; 3D contours reconstruction is obtained by marking the endocardium of both ventricles. (E and F) 4D echocardiographic videos showing P1 sham (E) and MI (F) hearts art at 21 dps throughout the cardiac cycle.
